# Unraveling the Multifaceted Roles of Extracellular Vesicles: Insights into Biology, Pharmacology, and Pharmaceutical Applications for Drug Delivery

**DOI:** 10.3390/ijms25010485

**Published:** 2023-12-29

**Authors:** Ali Al-Jipouri, Àuria Eritja, Milica Bozic

**Affiliations:** 1 Institute for Transfusion Medicine, University Hospital Essen, University of Duisburg-Essen, D-45147 Essen, Germany; ali.al-jipouri@uk-essen.de; 2 Vascular and Renal Translational Research Group, Biomedical Research Institute of Lleida Dr. Pifarré Foundation (IRBLLEIDA), 25196 Lleida, Spain; aeritja@irblleida.cat

**Keywords:** extracellular vesicles, lipid bilayer vesicles, cell-free therapeutics, pharmacokinetics, pharmacodynamics, pharmaceutical applications/drug delivery, nano-biocarriers

## Abstract

Extracellular vesicles (EVs) are nanoparticles released from various cell types that have emerged as powerful new therapeutic option for a variety of diseases. EVs are involved in the transmission of biological signals between cells and in the regulation of a variety of biological processes, highlighting them as potential novel targets/platforms for therapeutics intervention and/or delivery. Therefore, it is necessary to investigate new aspects of EVs’ biogenesis, biodistribution, metabolism, and excretion as well as safety/compatibility of both unmodified and engineered EVs upon administration in different pharmaceutical dosage forms and delivery systems. In this review, we summarize the current knowledge of essential physiological and pathological roles of EVs in different organs and organ systems. We provide an overview regarding application of EVs as therapeutic targets, therapeutics, and drug delivery platforms. We also explore various approaches implemented over the years to improve the dosage of specific EV products for different administration routes.

## 1. Introduction

Extracellular vesicles (EVs) are lipid bilayer vesicles released and taken up by diverse types of cells, thus playing an important role in different physiological and pathological processes. In a physiological context, EVs serve as facilitators of intercellular communication, whereas in pathological circumstances, they play roles in the onset, aggravation, and resilience of various diseases [1]. Owing to their distinctive attributes, EVs hold significant promise in formulating innovative therapeutic approaches for various diseases.

Comprehension of the regulatory mechanisms of EVs in diverse biological processes and intercellular communication is essential for unlocking their clinical potential and broadening their applications. Various non-clinical trials aim to provide comprehensive data on the pharmacodynamics, pharmacokinetics, and toxicity of EV products to support their investigation in future clinical trials, focusing on their efficacy and safety. The therapeutic trinity concept of EVs encompasses three primary therapeutic applications: (a) EVs as therapeutic targets; (b) EVs as therapeutics; and (c) EVs as drug delivery platforms. 

The current review outlines important physiological roles of EVs in different organs and describes their crucial pathological roles in the development of several diseases. We provide an overview of the most recent discoveries regarding the three primary therapeutic uses of EVs: inhibiting their pathological functions for therapeutic targeting, leveraging their natural functions for therapeutic purposes, and employing their in vivo kinetics as a foundation for drug delivery platforms. Finally, we discuss various strategies that have been undertaken during recent decades to improve EV-based dosage forms for various routes of administration.

## 2. Biology of Extracellular Vesicles

### 2.1. Biogenesis of Extracellular Vesicles

Extracellular vesicles (EVs) are lipid bilayer vesicles composed of proteins, lipids, and nucleic acids which are heterogenous in structure and function [2]. EVs are released by cells from prokaryotes to lower/higher eukaryotes, and plants [3], and play an important role in mediating physiological and pathological processes [4,5,6,7] (Table 1). As per their biogenesis, EVs are divided into two main types: Exosomes and microvesicles [2,4]. While apoptotic bodies may also be considered a type of EV, their role in intercellular communication is less studied and will not be reviewed here. Exosomes (30–150 nm) originate through the invagination of the limiting membrane of the early endosome, i.e., multivesicular bodies (MVBs) and are released to the extracellular environment upon fusion of MVBs with the plasma membrane [5]. Microvesicles (MVs) (100–1000 nm) are produced by outward budding and pinching of the plasma membrane [8,9]. The intracellular membrane is not involved during the secretion of microvesicles, and thus the membrane composition closely mirrors that of parent cells, a key difference from exosomes, which are heavily enriched in phosphatidylserine [4]. Although several other types of EVs released from plasma membranes have been discovered, such as migrasomes, ciliary ectosomes, secreted midbody remnants, exophers, etc., they have recently been classified into two major categories: exosomes (originating from the endosomal compartment) and ectosomes (originating from the plasma membrane) [10]. Recently, several mechanisms have been identified to regulate the biogenesis of EVs, thereby facilitating the sorting of protein and RNA cargo to generate EVs with a precise biochemical composition [11,12]. EV contents, size, and membrane composition are highly heterogeneous, dynamic, and dependent on the cellular source, state, and environmental conditions. 

### 2.2. Composition of Extracellular Vesicles

The composition of EV subgroups varies greatly depending on their source and isolation or enrichment techniques. Exosomes’ protein topology is the same as that of the releasing cell plasma membrane due to fusion of MVBs with plasma membrane, whereas the protein topology of microvesicles is heterogenous due to the direct budding off-plasma membrane [4]. Because there are no specific markers that differentiate between exosomes and microvesicles, investigating these two groups individually remains challenging [10]. The content of EV proteins ranges from general EV markers, subdivided into exosome markers (tetraspanins (CD9, CD63, CD81, and CD82), syntenin-1, TSG101, and Alix) and microvesicle markers (glycoprotein 1b, actinin-4, heat shock protein (HSP) 90B1, and myosin light chain) to post-translational protein modifications that specifically reflect vesicle localization, cellular origin (tissue-specific proteins), and secretion machinery [13,14]. EVs are highly abundant in cytoskeletal, cytosolic, heat shock, plasma membrane, and vesicle trafficking proteins, while they are less abundant in intracellular organelle proteins [12]. Furthermore, lipids are major components of EVs and have important roles during EV biogenesis, release, targeting, and cellular uptake [15]. The lipid composition of EV membrane depends on the type and physio-pathological status of releasing cells and determines their biological properties. Compared to parental cells, EVs are enriched in sphingolipids (i.e., sphingomyelin and ceramides) and glycerophospholipids containing saturated fatty acids [16]. These lipids resemble rafts that are important for increasing EV membrane rigidity and stability in biological fluids compared to parental cells. Moreover, the phospholipids that make up the EV membrane are also precursors of bioactive molecules (i.e., lysophospholipids and eicosanoids) that are able to mediate several processes in target cells, such as immune signaling and inflammation [16].

**Table 1:** Selected organ-derived physiological “Good” versus pathological “Bad” EVs.

Homeostasis State	EVs Source	EVs Cargo	Physiological “Good” Effect	Pathological “Bad” Effect	Ref.
**Urinary Tract** Water-Salt Balance	Nephron collecting duct epithelial cell-derived EVs	AQP2 protein/miRNA	Balancing overall water and ion levels in response to blood osmolality	Diabetic nephropathy (AQP2-AQP5 interaction) and nephrogenic diabetes insipidus (AQP2 mutation) result in the inability to concentrate urine	[17]
**Gastrointestinal Tract** Gut–Brain–Microbiota Axis (GBMAx)	GIT-Microbiota (Bacteroidota—Gram negative/Firmicutes—Gram positive)-derived OMV/MV ratio	OMVs carrying LPS cross BBB	Low (Bacteroidota/Firmicutes)-derived OMV/MV ratio reduces GBMAx permeability, producing normal child brain development and function	High (Bacteroidota/Firmicutes)-derived OMV/MV ratio increases GBMAx permeability; therefore, children are vulnerable to ASDs	[18]
**Musculoskeletal System** Myogenesis	Muscle precursor satellite cell-derived EVs	MyomiR miR-206	Upregulation of miR-206 targets ribosome binding protein 1 required for collagen synthesis along with dystrophin, which stimulates asymmetric division of satellite cells and will help repair muscle injury and reduce extracellular matrix deposition ideal for muscle remodeling	DMD is caused by a mutation in the dystrophin gene. Therefore, upregulation of miR-206 will further promote collagen synthesis at the expense of quiescent satellite cells, inflammatory cytokine secretion, and disturb calcium/mitochondrial homeostasis, contributing to the replacement of muscles with fibrous and adipose tissues	[19]
**Reproductive Tract** Semi-Allogeneic Fetus Tolerance and Self- Recognition	STB-derived EVs	NKG2D receptor binding MIC-related proteins; A, B, and UL16, pro-apoptotic proteins; FASL and TRAIL	STB-EV MICs and pro-apoptotics maintain semi-allogeneic fetus immune tolerance by suppressing immunity at the fetal–maternal interface via downregulating NKG2D NK cells and promoting T_reg_ cell development through HSPEI and their miRNA cargo	STB-EVs carrying MICs can induce semi-allogeneic fetus rejection, i.e., miscarriage by cross-dressing maternal APCs, thereby activating NKG2D NK to attack fetal cells	[20]
**Central Nervous System** Sonic Hedgehog (SHH) Signaling Pathway	Cerebellar Purkinje Cell-derived AXL-RAB18-TMED10 (ART)-EVs	SHH protein	SHH protein stimulates proliferation of GCPs, a progenitor cell that generates granule neurons, the most abundant neuron in the brain	Loss-of-function (LOF) mutations in the ESCRT-III member, CHMP1A required for vesicular SHH secretion causes microcephaly with pontocerebellar hypoplasia and short stature in humans	[21]
**Cardiovascular System** Blood Coagulation	Platelet-derived EVs	TF-CD142	Platelet EVs mediate the homeostasis necessary for embryogenesis, angiogenesis, and inflammation	Human Scott syndrome is a mild bleeding disorder caused by loss of Ca^2+^-dependent scramblase activity. Upon vascular damage, the perivascular TF and not the platelet EVs’ TF will initiate the coagulation process	[22]
**Immune System** Immune Tolerance versus Immune Regulation	APCs: DCs, BLs, and MP-derived EVs	MHC-I and -II versus immunoregulatory molecules: PD-L1, CTLA4, FASL, and TRAIL	The participation of EVs in the cross-presentation of exogenous antigens on MHC-I complexes to CD8+ T cells has an important role in immunity against viruses and tumors and in the immune response upon vaccination and induction of tolerance	EVs express immunoregulatory molecules: PDL1, CTLA4, FASL, and TRAIL, which interact with cognate ligands and receptors expressed T and NK cells, inhibit their activity, or induce apoptosis	[20]

Aquaporin-2 (AQP2); outer membrane vesicles (OMVs)/membrane vesicles (MVs); lipopolysaccharides (LPS); blood–brain barrier (BBB); autism spectrum disorders (ASDs); Duchenne muscular dystrophy (DMD); Syncytiotrophoblast (STB); natural killer group 2 member D (NKG2D); major histocompatibility complex class-I chain (MIC); antigen presenting cells (APCs); granule cell precursors (GCPs); endosomal sorting complexes required for transport (ESCRT); charged multivesicular body protein 1A (CHMP1A); tissue factor (TF); dendritic cells (DCs); B lymphocytes (BLs); macrophages (MPs); major histocompatibility complexes (MHCs); programmed cell death ligand 1 (PD-L1); cytotoxic T-lymphocyte-associated antigen 4 (CTLA4).

### 2.3. Physiological Roles of Extracellular Vesicles

EVs are produced and taken up by all types of cells. Therefore, substances that make EVs or are enclosed within them represent physiological components of the body. Cells rely on their secretome, more specifically on EVs, to induce various biological effects and physiological functions [23,24]. Furthermore, as EVs can sum up a large part of the parental cell’s biological effects, they are considered potential therapeutic agents. Indeed, preclinical studies have shown beneficial effects of EVs/secretome from various cell sources to treat many injuries of the heart, kidney, liver, brain, and skin [25,26,27,28,29]. EVs exert their basic physiological functions in a pleiotropic manner via (a) protein-/lipid-ligands’ direct cell surface receptor activation, and/or (b) recipient cell plasma membrane fusion and delivery of effectors (proteins and nucleic acids) [30,31,32,33], playing critical roles in stem cell maintenance [34], tissue repair [35], immunosurveillance [20], and blood coagulation [36]. Body fluid (urine, saliva, synovial, bile, cerebrospinal, bronchoalveolar, nasal, uterine, amniotic, breast, blood, feces, and seminal)-derived EVs are a mixture of vesicles that originate from various sources, such as cells in body fluids and/or cells that line extruded body fluid cavities. Thus, the contents of these EVs act as a source of physiological and pathological information, which can be transmitted over a long distance. In this section, we will focus on important physiological roles of EVs in maintaining the homeostasis of different organs.

#### 2.3.1. Urinary Tract

It has recently been shown that 3% of the total urinary protein content originates from EVs [37]. Although urinary EVs serve as a reservoir of biomarkers that come from the kidneys, ureters, urinary bladder, urethra [38], and prostate [39], their secretion and reuptake are essential in intercellular communication along the nephron and thus preservation of homeostasis of the urinary tract [40]. To differentiate between kidney-derived EVs and other infiltrating EVs, CD24 and CD133 may be of interest as kidney-specific urinary EV markers [41]. In the healthy organism, EVs contribute to the regulation of water–salt balance, where vasopressin-mediated water channel aquaporin-2 (AQP2), an apical Na^+^ transporter protein carried by EVs to the collecting duct cells [42], plays an important role. Thus, EVs control AQP2 trafficking and fusion with the apical plasma membrane, increasing nephron water permeability and hence water retention within the kidney [17]. Similarly, the direct action of one of EVs’ resident proteins, the angiotensin-converting enzyme (ACE) of the renin–angiotensin system (RAS), plays a role in water homeostasis [43]. Additionally, urinary EVs are rich in innate immune effectors (calprotectin and lysozyme C) that contribute to host defense within the urinary tract [44]. Moreover, urinary EVs expressing tissue factor (TF) can promote coagulation and hemostasis and thus reduce blood loss and contribute to host defense by reducing the risk of microorganisms entering the body through urinary and urethral epithelia [45]. 

#### 2.3.2. Gastrointestinal Tract 

Saliva is the most easily accessible biofluid and is considered as a mirror of general health. It is currently considered as a biofluid suitable for multilevel assessments [46]. Saliva-derived EVs are biologically active due to their protein and nucleic acid content, and, upon internalization by oral keratinocytes and macrophages, induce alterations in gene expression [47,48]. The source of saliva-derived EVs is the epithelial cells lining the salivary glands, as well as granulocytes found in saliva [49]. Saliva-derived EVs from healthy subjects have been shown to contain TF and CD26. The former can initiate blood coagulation (plasma-free clotting) [50], whereas the latter can cleave substance P and chemokines [51]. The bulk of the human microbiota inhabits the gastrointestinal tract (GIT), where it modulates diverse aspects such as insulin signaling, behavior, and allergy [52,53,54,55]. Similar to EVs that play a role in the host’s intercellular communication, microbiota release outer membrane vesicles (OMVs) that play a role in host–microbiota communication [56]. Diet and lifestyle influence microbiota and thus GIT homeostasis, which is highly dependent on inter-microbial communication, as well as host–microbial communication. Compared to eukaryotic EVs, OMVs are slightly smaller, ranging between 20 and 400 nm [57], and yet their physicochemical characteristics are similar. Nevertheless, the exact physiological role of OMVs is currently unclear. GIT homeostasis depends on healthy competition of microbiota with each other, and host neutralization of pathogenic lipopolysaccharide (LPS) components. This balance is necessary to maintain proper energy levels, lipid homeostasis, inflammatory homeostasis, and an effective GIT–blood barrier (GBB). EVs from *Akkermansia muciniphila* (Am), a beneficial bacterium that alleviates gut barrier disruption, have been found in fecal samples of healthy subjects, while in vitro and in vivo studies have shown their beneficial effect in intestinal barrier integrity [58].

#### 2.3.3. Musculoskeletal System

The musculoskeletal system provides structure for the body; thus, intercellular communication is vital for maintaining bone and muscle homeostasis, as well as for the regeneration after injury. EVs derived from skeletal muscle carry myokines, proteins, miRNA, and mRNA that are essential for the preservation of muscular homeostasis, development, and myogenesis [59,60,61]. Skeletal muscle is highly enriched in miRNAs (MyomiRs), such as miR-1, miR-133, and miR-206 [62]; hence, EVs that carry these miRNAs play a role in controlling myogenic homeostasis, proliferation, and differentiation, especially after injury and during exercise [63,64]. Muscle-derived EVs containing miR-16 that is taken up by the pancreas can modulate beta-cell proliferation and insulin secretion to regulate metabolism [65]. Furthermore, muscle-derived EVs carrying miR-206 regulate extracellular matrix collagen expression that facilitates fiber growth during repair [66]. Although the communication between bone-forming osteoblasts and bone-recycling osteoclasts is well-documented [67], the role of EVs participating in these processes, as well as in the synovial fluid production locally, has recently become an area of interest. Namely, primary bone marrow-derived mast cell EVs carrying mRNA and miRNA were able to drive protein production in recipient mast cells [68]. Furthermore, osteoblast-derived EVs transported the receptor activator of nuclear factor kappa-B ligand (RANKL) to osteoclast precursors, facilitating their formation in vitro [69]. It has been reported that mouse osteoclast-derived EVs carrying RANKL inhibited secretion of 1,25-dihydroxyvitamin D3, which regulated the formation of new osteoclasts [70]. The natural aging process is accompanied by bone deterioration, with EVs having a role in this process. Namely, EVs derived from the plasma of older adults have been shown to inhibit osteogenesis, mostly due to a decrease in Galectin-3 expression, which contributed to age-related loss of capacity for osteogenic differentiation [71]. Conversion of cartilage into a mineralized bone is a process mediated by EVs derived from calcified tissues carrying ossifying enzymes [72,73]. Thus, these enzymes mediate the local increment in orthophosphate that drives hydroxyapatite formation at matrix EV accumulation sites [73]. Therefore, a change in EVs during the aging process leads to the change in cargo and surface proteins, which may lead to functional changes.

#### 2.3.4. Reproductive Tract 

EVs have important roles during all stages of reproduction, starting from sperm and egg development, through fertilization and implantation, to maternal–fetal communication later in pregnancy [74]. It has been reported that the fusion of the prostasome (small vesicles secreted by the prostate) with sperm leads to an increased sperm motility, which is regulated by intracellular Ca^2+^ [75]. The process begins with prostasomes transferring CD38 into sperm and stimulating cyclic ADP-ribose (cADPR) production. In addition, prostasomes facilitate sperm–oocyte fusion, protect sperm from lysis [76], and have antibacterial activity [77]. Of note, luminal fluid EVs from mouse oviduct transported Ca^2+^-ATPase 4a (PMCA4) into sperm [78], triggering sperm motility and facilitating sperm–oocyte fusion required for fertilization [79]. Indeed, during incubation of CD9−/− sperm with CD9−/− eggs, the fusion was enabled only by the addition of EVs derived from CD9+/+ eggs. Furthermore, anti-CD9 mAb blocked the binding of sperm with CD9-containing vesicles, preventing sperm–egg fusion [80]. 

In addition to preconception, fertilization, and implantation, EVs have also been shown to play key roles during pregnancy, specifically in early trophoblast development and maternal–fetal and amniotic fluid signaling. Namely, extravillous trophoblast (EVT)-EVs express human leukocyte antigen G “HLA-G” and have been shown to be present in the maternal circulation from the first trimester of pregnancy [81]. Although EVT-EVs confer maternal tolerance and adaptation to pregnancy, their immunomodulatory properties ensure uterine tolerance to foreign antigens presented by the developing fetus. In addition, placenta-derived EVs have been shown to reduce cytotoxicity of CD4+, CD8+, and NK cells through Ig-like receptors and the NKG2D NK cell receptors, respectively [82]. During the late stages of pregnancy, syncytiotrophoblast (STB)-EVs are released directly into the maternal circulation and can be uniquely recognized by a placental alkaline phosphatase [83]. STB-EVs have been shown to carry several proteins including endoglin, plasminogen activator inhibitor, soluble fms-like kinase (sFlt), and endothelial nitric oxide synthase [84,85], as well as miRNAs [86,87], tRNA [88], and DNA [81,89]. The physiological functions of peripherally circulating STB-EVs in both in vitro and in vivo investigations are broad and their molecular contents as well as surface markers are powerful and cannot be underestimated. 

#### 2.3.5. Central Nervous System 

The brain is characterized by diverse and dynamic cell populations such as neurons, astrocytes, microglia, oligodendrocytes, and vascular cells. Since all cells of the central nervous system (CNS) release EVs, the neurovascular unit requires an efficient process of intercellular communication. It has been shown that increased neural activity is associated with increased release of EVs [90], a process vital for the efficient maintenance of synapses. This EV-driven neuronal activity is especially important during development, when activity-dependent pruning of synapses forms part of normal brain development [91]. In addition, intercellular communication also plays a key role in the regional development of the CNS, e.g., sonic hedgehog (shh) signaling, known to regulate cortical development [92]. Thus, shh signaling is regulated by differential expression accessory molecules on the surface of different EV populations. In the adult CNS, there are ongoing and essential interactions between cells that must be maintained for the CNS to function normally, as microglia need to remain in a quiescent/observable state, the blood–brain barrier (BBB) must remain intact, and astrocytes must maintain healthy functionality. The microglia’s quiescent state stems from the interaction between CX3CL1 (fractalkine “find me” signal) on the neuron with its receptor CX3CL1 on microglia, thereby reducing proinflammatory cytokine release (IL-6, IL-1β, and TNFα) and increasing anti-inflammatory cytokine release (IL-10), with an increased ratio of p-AMPK/AMPK and expression of Nrf2 after germinal matrix hemorrhage (GMH) [93]. Similarly, in the immune system, endothelial cell-derived EVs bear fractalkine on their surface to attract CX3CL1^+^ monocytes, acting as homing signals [94]. The BBB consists of a unit of cells including pericytes, astrocytes, and endothelial cells, the latter of which are connected via tight junctions preventing the normal migration of cells and macromolecules that appear in the fenestrated vasculature of the peripheral circulation [95]. The potential of EVs to cross the BBB to deliver drugs [96], passing from periphery to brain [97], and from brain to periphery [98], makes them an effective means of communication across the intact BBB. For example, EVs derived from brain pericytes are pro-angiogenic and have a role in regulation normal growth and function at the BBB [99]. Although the complexity of the brain requires reductionist approaches, the majority of EV release mechanisms at the BBB have been implemented in cell culture. 

#### 2.3.6. Cardiovascular System (Blood Pressure and Coagulation)

The vascular system maintains blood pressure by generating and releasing vasoactive chemicals. Enrichment of EVs with angiotensin II type I receptors can alter blood pressure [100], whereas EV-mediated inhibition of endothelial nitric oxide synthase (eNOS) could play a role in regulating nitric oxide (NO) production [101]. Coagulation is an important and dynamic process that maintains the integrity of the circulatory system, where platelet clumping plays the main role. It has been demonstrated that the presence of activated platelet-derived phospholipid-rich vesicles (EVs) in plasma mediates blood clotting [102]. Some conditions such as exercise, hypoxia, inflammation, and consumption of a high-fat diet increase the level of circulating platelet EVs [103]. Similar to EVs in general, platelet EVs can be classified based on their sizes and molecular contents into different subpopulations [104]. Platelet EVs range in size from large EVs (microparticles) [105], tubular elongated EVs [106], to smaller EVs (70–150 nm) resembling exosomes. Their load includes proteins from the plasma membrane, cytosol, organelles, adhesion receptors, coagulation and transcription factors, growth factors, active enzymes, cytokines, and chemokines [105]. Also, the main mediators of interaction with circulatory cells and matrices (fibrin) include GP IIa/IIIa (CD41/CD61), GP Ib (CD42b), P-selectin (CD62P), and CD40L (CD154), as well as unique exosomal markers (CD9, CD63, CD81, HSP70, TSG101 [103]). The content of platelet EVs can be confusing, as factors with opposing functions, e.g., pro- and anti-coagulant substances, can be detected [107]. In addition, they contain small metabolites [108], and RNAome comprises microRNAs (miRNAs), YRNAs, and circularRNAs (ciRNAs) [31] originating from parent megakaryocytes. Although the lifespan of platelets is about 10 days, exogenously injected EVs are cleared from the circulation within 10–60 min [103]. The most unique physiological role of platelet EVs is their ability to promote coagulation and thus participate in hemostasis. Human Scott syndrome abolishes platelet phosphatidylserine (PS) exposure, microvesiculation (i.e., EV formation), and thrombosis, and results in a mild bleeding disorder [22]. Upon platelet activation, disruption of the membrane phospholipid bilayer resulted in impaired PS externalization and decreased procoagulant activity, abolishing fibrin formation at sites of vascular damage [109]. The relationship between membrane phospholipid bilayer scrambling and EV formation in human Scott syndrome was found to be due to a gene defect encoding the transmembrane protein 16F (TMEM16F), an occult Ca^2+^-activated phospholipid scramblase (CaPLSase) that passively transports phospholipids down their chemical gradients and mediates blood coagulation [110]. Physiological hypoxic exercise training increased the level of pro-coagulant EVs and thus increased thrombin production [111]. Larger EVs (microparticles) from different cellular sources, rather than exosomes [112], show pro-coagulant activity in relation to tissue factor (TF^+^) EVs [113], which also act in other body fluids such as saliva and urine [114]. 

#### 2.3.7. Immune System 

Cell-to-cell communication is an essential aspect of an efficient immune system capable of protecting the host from injury, infection, and disease. While soluble factors such as chemokines and cytokines are known to modulate the immune system, EVs have been identified as pivotal players in the initiation and resolution of inflammation [1]. EV signaling plays a key role in the innate immune response to injury or infection [115,116]. EVs derived from neutrophils, monocytes, and macrophages are released upon stimulation by inflammatory and damage mediators and/or pathogen-associated molecular patterns [117,118]. It has been demonstrated that patients with inflammatory and infectious diseases have an increased number of circulating EVs derived from immune cells [119,120] that contribute to the restoration of homeostasis. In addition to antimicrobial effects of neutrophil-derived EVs [121], they enhance the immunological role of their parent cells by increasing the expression of IL-6 and ICAM-1 on endothelial cells [122], thereby facilitating their migration across the endothelial barrier. These EVs are enriched with numerous chemokines that direct leukocytes to the site of inflammation [115,123]. In contrast, cancer cell-derived EVs taken up by leukocytes can also trigger a response [124], confirming a bidirectional communication pathway. Despite the lack of in vivo evidence, investigating the role of EVs in immune cell activation is currently considered one of the most important areas of research in the field of EV biology. Raposo et al., 1996 demonstrated that Epstein–Barr Virus (EBV)-infected B-cell line released EVs that were able to stimulate T-cell proliferation and an antigen-specific response [125]. This immune stimulation was attributed to EVs that served as antigen-presenting vessels, as they were enriched for Major Histocompatibility Complex (MHC) II and EBV-specific proteins. A similar earlier study showed that upon activation, dendritic cells (DCs) secrete antigen-presenting EVs, enriched with MHC complexes and T-cell co-stimulatory molecules that prime a T-cell-specific cytotoxic response with higher immunogenicity [126]. Subsequent studies showed that EVs expressed MHC class I and II molecules, and adhesion and co-stimulatory molecules were able directly to stimulate CD8+ and CD4+ T-cells via binding their respective plasma membrane receptors [127]. Moreover, a comparative study showed that DCs pulsed with tumor peptides released EVs that could induce a stronger anti-tumor CD4+ T-cell response than T-cells incubated with the peptides alone, which is evidence for the theory of enhanced immunity [128]. 

In terms of homeostatic ability, while some EVs are able to activate the immune system, other EVs are able to suppress the immune system. For example, natural killer-derived EVs (NK-EVs) show their cytotoxic activity only on activated, but not resting cells [129]. The suppressive effect of EVs is important during pregnancy, as the pro-inflammatory state can be harmful. Placental EVs are shed in large quantities during pregnancy [130,131] and have been associated with TNF-family ligands FasL and TRAIL, leading to apoptosis in activated lymphocytes [132,133]. In the same context, FasL plus plasma EVs have been shown to induce apoptosis of CD4+ T-cells [134]. Immunomodulatory EVs play an important role in the prevention of autoimmunity and chronic inflammation. The term tolerosomes was coined to demonstrate EV-mediated immune modulation by epithelial cells [135], which represents the key to the effective development of an allergic response. The same was demonstrated for EVs isolated from bronchoalveolar lavage fluid (BALF) of mice immunized against olive pollen allergen [136], and adoptively transferred into naïve mice. Immunized mice upon exposure to the allergen showed suppression of the immune response and production of Th2 cytokines. Similarly, responding to ovalbumin-loaded dendritic cell (DC)-derived EVs from MHC−/− mice stimulated antigen-specific T cells at the same magnitude as wild type EVs, i.e., MHC-independent immune response [137]. This stimulation of immune tolerance has been exploited therapeutically in diseases such as post-transplant graft vs. host [138]. 

### 2.4. Pathological Roles of Extracellular Vesicles

EVs can be enriched for pathogenic proteins and nucleic acids [139,140]; thus, biofluid analysis improves our understanding of their pathophysiological roles in different organs. In this section, we will focus on pathological roles of EVs in the development of several diseases, and we will explore their potential as biomarkers, as well as tools for potential therapeutic intervention. 

#### 2.4.1. Urinary Tract

Compared with other biological fluids, urine is widely available and relatively easy to obtain in large quantities, and thus the study of the functional role of EVs in kidney disease processes is a more accessible area than other areas of EV biology. This accessibility has made urinary EVs the most studied biomarkers of kidney and urinary tract diseases [141]. The functional unit of the kidney is the nephron, which begins with the glomerulus that filters the blood, towards the tubules and the collecting duct that regulates the composition of the urine into the tubular interstitial system. The involvement of EVs in a range of intrinsic kidney diseases has been well-established and affects all parts of the nephron, including glomerular and tubular injury, nephritis, fibrosis, and ion-channels and water transport disorders [142,143,144,145]. Although intra-glomerular communication within the glomerular vasculature has already been investigated in vasculitis, intra-nephron communication is challenging to study. In the plasma of patients with vasculitis, leukocyte EVs transferred B1-kinin receptors to glomerular endothelial cells, and promoted kinin-associated inflammation [143]. This demonstrates the bidirectional nature of EV communication between the circulatory system and urinary system. In addition, EVs have been shown to contribute to the outcome of glomerular–tubular inter-communication in disease progression [146]. Indeed, tubular epithelial cell EVs loaded with functional cargoes such as chemokines, TGF-β1, CCL2 mRNA, and osteopontin transferred these cargoes to interstitial macrophages, aggravating kidney damage [145]. In kidney diseases such as IgA nephropathy [147] and diabetic kidney disease [148], increased inflammation leads to cellular oxidative damage. The spread of inflammation between tubules and renal interstitium may lead to significant kidney damage. Indeed, EVs derived from TGF-β1-treated tubular cells exacerbated kidney injury and fibrosis [144]. This result was supported by the demonstration that EVs generated in vivo from animal kidney tissue with ischemia reperfusion injury transferred TGF-β1 into fibroblasts in vitro [149]. Besides communication between vasculature and glomerulus, as well as tubules and interstitial renal compartments, communication between proximal and distal tubular cells has also been demonstrated in vitro. For example, proximal tubule EVs have been shown to reduce sodium channel function in distal cells by transferring nucleic acid [150,151]. 

#### 2.4.2. Gastrointestinal Tract

As previously mentioned, microbiota release outer membrane vesicles (OMVs) that play local and systemic roles in maintaining homeostasis. Similarly, OMVs can also be released by harmful bacteria, such as the Gram-negative proteobacterium *Helicobacter pylorus* (Hp), whose chronic infection leads to chronic inflammation and the development of gastric cancer [152,153]. Hp OMVs have been shown to carry virulence genes (CagA and VacA) of the parent bacteria, which can induce the production of immunomodulatory cytokines in host macrophages and gastric epithelial cells [154,155]. As seen from in vitro and in vivo studies, Cy7 labeled Hp OMVs were able to infiltrate the gastric epithelium and still be detectable even 24 h after injection, causing inflammation and contributing to the development of gastric cancer [156]. It was later found that the inter-communication between Hp-infected gastric cancer cells and macrophages involved the internalization of EVs enriched with phosphorylated active growth factor isoform by macrophages in vitro and in vivo [157]. The apparent role of growth factor internalization is to educate macrophages toward a pro-tumorigenic phenotype, including an increase in IL-1β secretion that promotes tumor growth and progression in vivo [158]. These studies reveal the importance of the role of Hp OMVs in the development of gastric cancer and shed light on the diversity of OMVs derived from microorganisms. In addition to local effects, the gut also communicates with other parts of the body, such as the gut–brain–microbiota axis (GBMAx), which is an important player in psychiatric diseases [159]. During childhood, increased GBMAx permeability may lead to autism spectrum disorders (ASDs) [160,161], with microbiota by-products including EVs entering the circulation and interfering with normal development [162]. Profiling urinary bacterial OMVs in ASDs, with the aim of prophylaxis for children with pre- and pro-biotics could definitely be used to combat differences in microbial diversity [163]. Pathophysiologically, EVs derived from the gut microbiome may contribute directly or indirectly to a number of mental health disorders.

#### 2.4.3. Musculoskeletal System

Pathologies of the musculoskeletal system are particularly harmful for the body, while the accompanied pain and lack of movement are significant burdens for the healthcare system. It requires new intervention strategies in order to understand how EVs participate in the formation and strengthening of the musculoskeletal system. Various diseases, especially cancer, chronic infections, and heart failure, often cause Cachexia syndrome, a complex disease characterized by the loss of skeletal muscle and adipose tissue. About 50–80% of cancer patients suffer from Cachexia syndrome and weight loss with increased levels of circulating EVs [164]. Tumor EVs contain two markers, HSP70 and HSP90, which have been shown to effectively induce muscle wasting [165]. Although circulating EV contents and function in Duchenne muscular dystrophy (DMD) are still under investigation [19], in terms of biomarkers and therapeutics, they may assist in the early diagnosis [166]. Recently, it was shown that GW4869 (neutral sphingomyelinase (nSMase) inhibitor), which reduces EV release in mdx mice (DMD model), was protective against cardiac stress, which has been attributed to miRNA load [19]. In this sense, KO of the nSMase2/Smpd3 gene in these mice reduced muscle inflammation and improved functional performance [167]. Diseases of the skeletal system such as osteological tumors, chondrocytic disease, and inflammation influence EV populations within the circulation and their downstream effects on cells and organ systems. For example, synovial fibroblasts from patients with rheumatoid arthritis (RA) produce EVs containing the inflammatory protein TNF-α and stimulate NFkB production [168]. In contrast, synovial fluid EVs from men and women with OA showed an ability to reduce cellular metabolic activity [169]. These studies include the identification of the miRNA content of EVs that have shown enrichment of targeting of sex-specific signaling pathways and shed light on the importance of combining clinical data with pre-clinical research. 

#### 2.4.4. Reproductive Tract

In contrast to the role of EVs in normal gynecological processes, dysfunctional EV signaling has been associated with gynecological pathology and diseases occurring during pregnancy. These include endometriosis [170], polycystic ovary syndrome (PCOS) [171], pre-eclampsia [172], and gestational diabetes [173]. For example, elevated EV-associated RNA (DENNDIA gene) in a woman’s urine indicates polycystic ovary syndrome (PCOS) [174]. EV miRNAs involved in estradiol regulation were found to be downregulated; thus, EVs play a role in the initiation of PCOS [175]. Furthermore, PCOS is associated with a prothrombotic state, as affected women have been shown to have abundant amounts of platelet-derived EVs in their plasma, and this contributed to the higher cardiovascular risk in these patients [176,177]. Worldwide, pre-eclampsia is a leading cause of maternal and fetal morbidity and mortality [178], showing higher levels of circulating STB-EVs [179]. It has been found that placental EVs of pregnant women with pre-eclampsia can cause hypertension when injected into non-pregnant mice [180]. Moreover, 12 STB-EV-isolated miRNAs in peripheral plasma can be used to differentiate normal pregnancies from pre-eclampsia [181]. Physiologically, normal pregnancy represents a state of relative insulin resistance, while in some women, insulin resistance becomes pathological, leading to a disease called gestational diabetes mellitus (GDM). The level of EVs in maternal plasma is increased in GDM pregnancies compared to normal pregnancies [182,183]. Similarly, EVs derived from plasma from women with GDM induced glucose tolerance in non-pregnant mice [182]. These data demonstrate the powerful role that EVs play in maintaining reproductive health and the development of a successful and healthy pregnancy. 

#### 2.4.5. Central Nervous System 

Diseases of the CNS can be divided into acute injuries such as stroke and trauma, and chronic neurodegenerative diseases such as Parkinson’s disease. Acute trauma injury causes rapid mechanical damage to blood vessels, neurons, and glia, leading to the death of multiple cell types, as well as the release of damage-associated molecular patterns. In contrast, neurodegenerative diseases are more limited to single cell types, before becoming more widespread. Therefore, the role that EV signaling plays in these pathologies depends a great deal on the characteristics of the disease. It has been demonstrated that traumatic brain injury in mice caused a release of EVs from microglia, while injection of these EVs into a healthy mouse brain led to neuroinflammation [184]. Intrastriatal injection of brain-derived EVs has been reported to activate microglia and stimulate the release of pro-inflammatory mediators; therefore, EVs could act locally to exacerbate central inflammation after initial injury [185]. Of note, it has been demonstrated that after stroke [186] and TBI [187,188], an increased number of circulating EVs had the potential to induce systemic immune reaction [186,188], indicating EV-mediated brain–immune system communication. For example, the multiple sclerosis (MS) relapse group demonstrated an increase in circulating myeloid^+^ EVs as potential markers of neuroinflammatory attacks [189]. Thus, the increment in myeloid-derived EVs in an MS patient’s cerebrospinal fluid (CSF) depends on the patient’s condition (stable vs. acute MS) and is related to the number of neuroinflammatory lesions [189]. On the other hand, in chronic CNS diseases such as Parkinson’s and Alzheimer’s, characterized by a slow degeneration of neurons over a number of years [190], EVs could propagate the disease. In a mouse model of Alzheimer’s disease [191], as well as aging [192], the propagation of tau fibrils has been shown to be mediated by EVs. Recent work has demonstrated that familial Alzheimer’s-induced pluripotent stem cell (IPSC)-derived EVs are able to induce tau pathology in naïve mice [193]. In Parkinson’s patients, EVs have been proposed as a link for α-synuclein aggregation, as they also have the potential to be used as diagnostic tools for stratifying patients [194,195]. 

#### 2.4.6. Cardiovascular System (Blood Pressure and Coagulation)

EVs have been shown to participate in the pathogenesis of different cardiovascular diseases (CVDs) [196,197,198]. Direct contact between blood vessel endothelial cells and peripheral blood allows endothelial cell (EC)-EVs to be rapidly released and delivered to distant organs, thus influencing the onset of different CVDs. Although EVs may also have downstream pathogenic consequences, they have been shown to have a hemostatic blood pressure modulating effect. In rats, circulating EVs have been shown to be able to suppress vasodilation [199,200] and thus play an important role in hypertension. Namely, it has been shown that hypertensive patients show an increase in EC-EVs in the plasma [201] and urine [202]. Thus, blocking the generation and release of EVs was found to reduce blood pressure in hypertensive rats [203]. Furthermore, an increase in circulatory EC-EV levels has been found in patients with endothelial dysfunction [204], obstructive sleep apnea [205], obesity [206,207], renal failure [208], coronary artery disease [209], myocardial infarction [210,211,212], β-thalassemia [213], and stroke [214]. Nevertheless, it is not clear if the increase in their number contributes to these conditions. 

Although the coagulation process is a homeostatic response to injury, inappropriate platelet activation can lead to pathological thrombosis and atherosclerosis [215]. It has been estimated that platelet EVs account for 25% of the procoagulant/anticoagulant activity in blood [216] and exhibit 50–100-fold higher procoagulant activity than the surface of activated platelets [217]. Thus, blocking the generation and release of platelet EVs has been found to lead to an increase in abnormal bleeding [218]. Fibrin fibers have been shown to contain EV-positive CD61, which plays a role in platelet aggregation [219]. Platelet EVs provided pro-hemostasis support during uncontrolled bleeding by modulating the kinetics of fibrin formation, clot structure, as well as fibrinolysis, thus preventing hemorrhagic shock [220]. 

Studies on the role of platelet EVs related to inflammation have been reported to increase their numbers in response to infection with viruses and parasites [221]. Platelet EVs orchestrate the immune response by modulating the performance of other immune cells [222]. Certainly, platelet EVs generated during inflammation [223] and acute liver injury and infiltrating into the bone marrow were essential for the regulation of megakaryocyte formation [224]. The results were corroborated in human bone marrow biopsies [223]. This demonstrates that platelet EV proxies mediate their pathological functions, thus hindering the identification of their roles in vivo. Implications for platelet EVs in autoimmunity [225], e.g., in RA, have shown that platelet EVs contain IL-1α and IL-1β in synovial fluid. These EVs are triggered by collagen receptor GPVI and promoted synovial cell activation and production of IL-6 and IL-8 (CINC-1) [226]. It has also been reported in RA that peripheral blood-derived regulatory T-cells transduced with platelet EVs ceased to differentiate into IL-17 and IFN-γ producing proinflammatory cells [227,228]. The role of platelet EVs in inflammation highlights the importance of studying them in a system of multiple cell types. 

#### 2.4.7. Immune System

Autoimmune or inflammatory diseases are involved in creating a pro-inflammatory environment that is associated with an increment in circulating EVs. Furthermore, based on the knowledge on the involvement of EVs in intercellular communication between cells of the immune system, inappropriate EV-mediated activation can contribute to pathological conditions. Thus, platelet EVs in the synovial fluid of RA patients is likely responsible for triggering an inflammatory reaction within the affected joints [229]. Similarly, in patients with inflammatory bowel disease, granulocyte-derived EVs enriched for metalloproteinases and pro-inflammatory cytokines have been shown to increase intestinal permeability [230]. Other studies have linked EV-mediated inflammation to CVDs [206,231,232,233,234], MS [189,235,236,237], and more. Autoimmune diseases can stem from EV-presenting self-antigens that auto-activate lymphocytes and trigger immune cells with antigen specificity for EVs’ own parent cells. Circulating EVs in systemic lupus erythematosus (SLE) that are enriched for antigenic DNA may act as an autoantigen to accelerate disease progression [238]. Similarly, the interaction between EVs and autoreactive T- and B-cells has been shown to trigger pancreatic inflammation and lead to the development of diabetes in non-obese diabetic (NOD) mice [239,240,241]. In contrast to activation of the immune system, EV-mediated suppression can be devastating. Currently, there is a large body of evidence showing the participation of EVs in the development of the metastatic niche by suppressing the circulating immune response to migrate tumor cells [242,243]. An active inflammatory response to cellular growth is a key mechanism to reduce tumor burden, which is currently being exploited, as EVs play an important role in this process. In vitro and in vivo studies showed that tumor-derived EVs expressing FasL and TRAIL activated regulatory T-cells and myeloid-derived suppressor cells (MDSCs), which prevented CD8+ T-cell from targeting the tumor [244]. FasL^+^ EVs were found in the sera of oral squamous cell carcinoma patients, and their level correlated with tumor burden and nodal involvement [245]. In addition, EVs suppress the immune system by reducing cytotoxic NK cells circulating in the lung and spleen, allowing metastatic niches to form in those organs. It has been demonstrated that neutrophil mobilization is required for tumor growth; thus, targeting EV release by GTPase RAB27A/B blockade in mice decreased primary mammary carcinoma tumor growth and its dissemination into the lung [246]. 

## 3. Pharmacology of Extracellular Vesicles 

**Figure 1:**
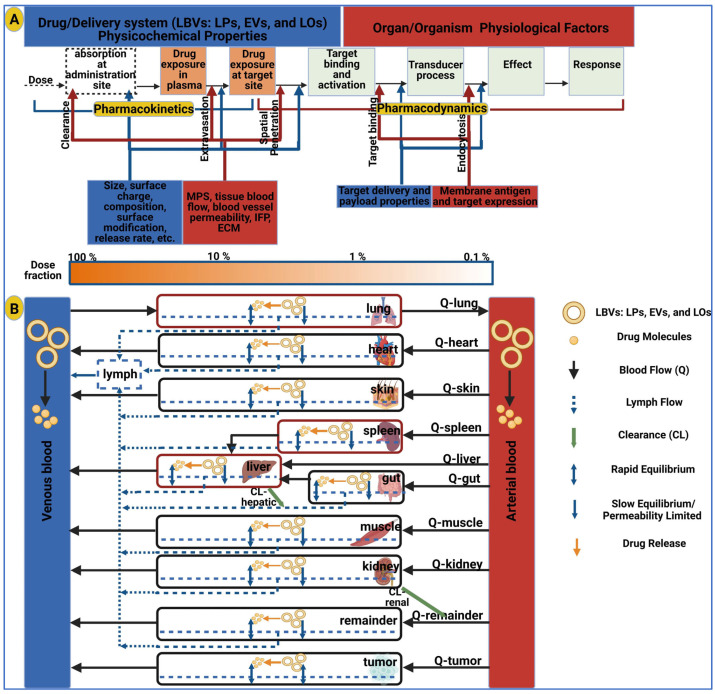
Schematic of pharmacokinetics–pharmacodynamics (PK-PD) modeling of the lipid bilayer vesicles (LBVs): liposome (LP), extracellular vesicle (EV), and hybrid liposoxome (LO) drug delivery system development. (**A**) PK-PD modeling connects the drug dose (encapsulated/engineered vesicles) to the physiological response, related to the drug delivery system properties and the physiological factors. A series of events describes the flow from administration, drug exposure (plasma and target site), receptor binding and activation, transduction to effect, and effect on physiological response. (**B**) Physiologically based PK (PBPK) modeling for nanodrugs (encapsulated/engineered vesicles) consists of LBVs and released small molecules. LBVs are linked via small orange arrows to drug release. LBV tissue distribution is convection-driven (unidirectional blue arrows) except in the tumor, where passive diffusion is the main distribution mechanism due of the high interstitial pressure. In contrast, the release of small molecules is tissue-specific (i.e., phagocytosis, low tumor pH, etc.), whereas drug distribution is bidirectional (blue arrows). Enhanced accumulation of LBVs in the lung, spleen, and liver is associated with the leaky vascular structures and sequestration of the mononuclear phagocytic system (MPS) (small thicker orange arrows) with the exception of the higher tumor accumulation of LBVs, attributable to the enhanced permeation and retention (EPR) effect. The model also includes the lymphatic system to recycle LBVs (blue dashed arrows) from the interstitial space (created with https://app.biorender.com/ (accessed on 25 December 2023)).

Pharmacology branches include pharmacodynamics (PD), which studies the action of the drug on the organism (primary intended effects and secondary unintended effects) [247], and pharmacokinetics (PK), which studies the effect of the organism on the drug related to absorption, distribution, metabolism, and elimination (ADME) [248]. The objective of non-clinical trials is to provide in vitro, ex vivo, and in vivo data on the PD, PK, and toxicity profile of the given EV product for the chosen route of administration in order to support its investigation in a clinical trial in terms of efficacy and safety [249]. Principally, the PD and PK studies are performed earlier in the development phase, while toxicity tests are performed later. EVs generated from non-pretreatment and genetically modified cells that do not contain any transgenic product belong to the category of biomedical products. In contrast, EVs generated from genetically modified cells containing a transgenic product are considered gene therapy products (GTPs), a subclass of advanced therapy medicinal products (ATMPs) [250]. The European Medicine Agency (EMA) supports the classification of ATMPs through the committee for advanced therapy (CAT) with the recommendation that recombinant RNA-containing EVs be considered as GTPs [251,252], as the effects are directly related to these molecules. In contrast, recombinant peptides or protein-containing EVs are products of biotechnology. The following sections will address pharmacological and pharmaceutical aspects of EVs that stem from their composition and PK-PD model similarities to liposomes (LPs) [253] (Figure 1), where both are lipid bilayer vesicles (LBVs).

### 3.1. Pharmacodynamics of Extracellular Vesicles

EVs are involved in diverse biological processes, such as cell motility [254,255,256], differentiation [257,258,259], proliferation [260,261], apoptosis [262,263], reprogramming [264,265,266], and immunity [267,268], through regulation of intercellular communication. Understanding the regulatory mechanisms of EVs in these biological processes and in intercellular communication is crucial for their clinical potential [269,270] and applications [271,272,273,274,275,276,277,278,279]. This section will focus on therapeutic applications of EVs as therapeutic targets by blocking their pathological roles, as well as their use as therapeutics by employing their physiological roles. The use of EVs as drug delivery platforms by utilizing their in vivo kinetics will be discussed under Section 4. Pharmaceutical Applications of Extracellular Vesicles.

#### 3.1.1. Therapeutic Targeting of Extracellular Vesicles

Physiologically, EVs are mediators of intercellular communication, while pathologically, they are involved in the initiation, exacerbation, and resistance in various diseases [280]. This leads to a large proportion of unsuccessful treatment regimens, specifically in cancer, due to the role of EVs in conferring resistance to cancer cells, via immune evasion and metastasis, as well as to chemotherapy [281,282]. These unfavorable pathological roles of EVs can be intercepted by targeting the three main parts of EVs machinery: biogenesis cargo sorting, release, and uptake [283]. For instance, to abrogate the suppressive effect of multiple myeloma (MM) cell-derived EVs on the cytotoxic activity of natural killer (NC) cells, two long-chain omega-3 polyunsaturated fatty acids, eicosapentaenoic acid (EPA) and docosahexaenoic acid (DHA), should be used at the biogenesis level [284]. Thus, pre-treatment of MM cell lines with either EPA or DHA could largely reverse EVs’ natural killer-suppressing effects, hindering their biogenesis [284]. Similarly, the pro-angiogenic effects of breast cancer cell-derived EVs can be nullified using DHA, which alters the cargo sorting of EVs in favor of anti-tumor effects [285,286]. To disrupt the release of EVs from parent cells, efforts have been made using bioinformatics methods to hypothetically screen a large number of approved drugs to identify those with potential EV release-inhibiting effects [287,288,289]. For example, the natural antibiotic Manumycin A has been shown to inhibit prostate cancer cell EV secretion by blocking the Ras/Raf/ERK1/2 and hnRNP H1 pathways in vitro [278,290]. Targeting cancer cell-derived EV-mediated shedding of NK cell inhibitory ligands, MICA and MICB, prevented these cells from evading the immune system in mice [291,292]. Likewise, inhibition of lung tumor cells’ kras-derived EVs could reverse the induced immunosuppression and chemoresistance [293]. In the same sense, targeting the biogenesis of OMVs from prokaryotic cells (e.g., extrinsic pathogens) has the same physiological and pathological significance as targeting host cell biogenesis of EVs. These results suggest that the above-described targeting methods may be of interest in developing new therapies for some bacterial diseases. 

#### 3.1.2. Extracellular Vesicles as Therapeutics

Different cell types, such as mesenchymal stromal cells (MSCs), specific tumor cells (TCs), and immune professional antigen presenting cells (APCs), such as dendritic cells (DCs), B lymphocytes (BLs), and macrophages (MPs), produce EVs that can be used as drugs without any specific manipulation of their contents and/or associated molecules [294,295]. Also, further education of EV-producing parent cells via genetic engineering or pharmacological treatments could add more potential over their untreated/engineered counterparts, because produced EVs can carry a cargo of interest that aligns with therapeutic goals. 

##### Mesenchymal Stromal Cell-Derived Extracellular Vesicles (MSC-EVs) 

Five decades ago, MSCs were first recognized for their pluripotent potential, and recent findings suggest their regenerative and modulatory properties [296]. MSCs are the source of a myriad of active molecules [297]. However, concerns about the safety of cell-based therapies still challenge the applicability of MSCs for use in humans [298]. Two mechanisms by which MSCs can exert their beneficial effects are direct cell-to-cell contact with their target cells, and by release of soluble factors (including EVs) as a means of modulating their targets remotely [299,300,301]. The anti-apoptotic, pro-angiogenic, anti-inflammatory, proliferative, and trophic nature of MSC-EVs provide potent intrinsic regenerative properties that have been demonstrated in numerous organs [302,303,304,305,306]. Thus, mouse bone marrow endothelial progenitor cell-derived EVs improved the hemodynamic status of murine models of myocardial infarction (MI), showing significant pro-angiogenic effects [307]. Bone marrow, adipose tissue, and umbilical cord MSC-EVs inhibit cardiomyocyte apoptosis and promote angiogenesis, thus improving cardiac function and protecting myocardium [308]. The mechanism by which natural umbilical cord MSC-EVs alleviate liver injury after ischemia/reperfusion is due to the binding of miR-20a and two upregulated genes, Beclin-1 and FAS 3′ UTRs, thereby inhibiting apoptosis [309]. Injecting MSC-EVs locally into a murine model of retinal detachment (RD) significantly reduces levels of inflammatory cytokines TNF-α and IL-1β, Atg5 cleavage, and apoptosis of photoreceptor cells, thereby preserving the normal structure of the retina [310]. The existence of miRNAs targeting TLR4/NF-kB within MSC-EVs suppresses inflammation associated with peripheral neuropathy in a murine diabetic model by decreasing the expression of inflammatory cytokines and regulating the ratio of M1 and M2 macrophages, thereby improving neurovascular architecture [311]. 

In contrast to the aforementioned natural pharmacodynamics of MSC-EVs, engineering EV-producing MSCs leverages them towards producing stronger specialized EVs. The methods that can be employed range from stimulating EV-producing cells with, e.g., drugs, cytokines, growth factors, altering cell culture conditions (e.g., hypoxic vs. normoxic, 3D vs. 2D culture), and genetic engineering with genetic constructs (e.g., plasmids) [312]. The goals of these methods may be to increase the amount(s) of a specific molecule(s) in the EVs, alter the sorting of small RNAs, or even add/delete a specific gene in the final structure of the released EVs. Natural regenerative effects of human adipose tissue derived-MSC secretome in the lungs have been revealed to include proteins and lipids essential for maintaining protease/anti-protease homeostasis and anti-microbial activity. In vitro stimulation of MSCs with dexamethasone and IL-1β along with starvation leads to an increment in Alpha-1 antitrypsin (AAT), the major elastase-inhibitory enzyme in the lung [313]. Although MSC-EVs carrying miR-20a partially alleviated liver IR-induced injuries in rats, boosting these EVs with mimics of this miRNA resulted in the complete alleviation of the injury [314]. Similarly, MSC-EVs transfected with a miR-20b-3p mimic reduced calcium oxalate accumulation in rat kidneys, with downregulation of oxalate-induced autophagy and inflammation as responsible therapeutic effects [315].

##### Tumor Cell-Derived Extracellular Vesicles (TC-EVs) 

TC-EVs are rich in immunogenic tumor antigens [316], which, when taken up by DCs, address antigen cross-presentation by complexing with major histocompatibility complex type 1 (MHC I) [317] to both T-helper (Th) and cytotoxic T lymphocytes (CTL), thereby creating a potent anti-tumor response [318,319,320]. There are two main groups of tumor antigens, namely tumor-specific antigens (TSAs) and tumor-associated antigens (TAAs). The former represents the unique antigens (neoantigens) [321] resulting from mutagenic events, while the latter is present on nonmalignant cells with changes in their expression [322,323]. The difficulties of TSAs to be identified make TAAs the most promising antitumor therapeutics and vaccines. Examples of TAAs are carcinoembryonic antigen (CEA), the transmembrane glycoprotein Mucin 1 (MUC1), and melanoma-associated antigen (MAGE), each of which ranges from overexpression to aberrant expression in specific tumor types. However, their use is limited due to their limited number and poor immunogenicity [324,325]. Furthermore, the reason for designing a single vaccine against several types of tumors is a myth due to the inability of shared/overlapping antigens to be cross-presented efficiently by APCs. Interestingly, TC-EVs were shown to contain shared tumor antigens that were efficiently cross-presented by DCs and lead to cancer rejection in mice [326]. The reason for this finding lies in the presence of a separate group of proteins critical for a robust anti-tumor response, the original tumor antigens, and the overexpression of specific molecules and receptors that are essential for antigen sampling by APCs [327]. In this sense, the use of TC-EVs as natural samples of tumor antigens may be a viable option for developing effective antigen-based immunotherapies. Although the components of TC-EVs are diverse, one of the many strategies is to employ TC-EVs in ex vivo maturation and induction of DCs to induce robust CTL responses rather than additional activation stimuli in DCs, as well as to combine different oncolytic peptides to increase coverage of designed therapies/vaccines [324]. Like MSCs, tumor cells can be primed to produce EVs with enhanced therapeutic activities that, when presented to the host directly, elicit effects of interest, such as targeting the delivery of specific molecules. The complex composition of TC-EVs could be a major drawback of this strategy, which could lead to undesirable effects. This can be solved by exploiting the surface properties of TC-EVs without undesirable effects to produce biomimetics in which the drug-carrying core is coated with the outer covering of the TC-EVs [328,329]. One study showed HepG2 and SKBR3 TC-EVs transfected with a therapeutic anti-miR-21 to activate synthetic gold iron oxide nanoparticles and harness their potential in tumor targeting [330]. Thus, the targeting behavior of TC-EVs depends on the type of cancer cell from which they were isolated. The larger amounts of TC-EVs produced by tumor cells compared to their non-malignant counterparts [331] and their targeting behaviors based on their parent cells show that TC-EVs may be unusual candidates for serving as novel targeting therapies [332]. 

##### Immune Cell-Derived Extracellular Vesicles (IC-EVs) 

IC-EVs include DC-EVs (dexosomes) [126,333], BL-EVs, and MP-EVs, which carry MHC I and II, required for antigen presentation, as well as necessary co-stimulatory molecules [20,125]. It has been shown that adhesion molecules that direct EVs towards effector cells such as CD11b, CD9, and lactadherin are also released upon selective enrichment by these immune cells [333,334]. MP-EVs are part of the immune system, and they are not trapped or eliminated by the phagocytic system; therefore, they are superior to other microparticles used for drug delivery [335]. It has been shown that DC-EVs pulsing with tumor peptides can be used as an effective non-cellular vaccine to prime CTLs in murine tumors [126]. The DC-EVs used in this study efficiently presented tumor antigens loaded to effector cells, which finally eradicated tumors in an animal model of P815-mastocytoma and TS/A-mammary carcinoma [336]. Although this study and several similar studies demonstrated the potential of cell-free DC-EV vaccines, drawbacks, such as difficulties in long-term storage and challenges with targeted delivery, limit their applicability [337]. In addition to the antigen presenting potential of MP-EVs, they express adhesion molecules, e.g., LFA-1, where overexpression of complementary adhesion molecules, e.g., ICAM-1, facilitates EV–target interaction. As inflammation is an inseparable component of many conditions, this natural feature of MP-EVs can be used to deliver targeted drugs to inflamed sites [338]. This knowledge supports further exploration of the physiological properties of IC-EVs as well as the study of less-studied NK cell-derived EVs (NK-EVs), which could open new windows for designing novel therapeutics.

##### Human Microbiome-Derived Extracellular Vesicles (HMB-EVs) 

Bacteria-derived EVs, on the basis of their source and biogenesis, can be classified into membrane vesicles (MVs), originating from the inner membranes of Gram-positive bacteria, and OMVs, released by Gram-negative bacteria [339,340]. The roles of HMB-EVs in host cell homeostasis range from nutrient sources to horizontal gene transfer, and even nucleic acid delivery [341]. *Lactobacillus acidophilus*-derived MVs contain bacteriocins capable of eliminating opportunistic pathogens in vitro [342]. Similarities between prokaryotic EVs and their paternal microbes regarding the interaction of pathogen-associated molecular patterns (PAMPs) with their corresponding pattern recognition receptors (PRRs) combined with natural adjuvant properties could lead to the same antimicrobial immune response as that of the pathogen itself [343,344]. EVs derived from *Staphylococcus aureus* as a vaccine candidate trigger immune responses via the toll-like receptor (TLR) pathway in *Staphylococcus aureus*-induced pneumonia in mice [344]. Although microbial EV lipopolysaccharide (LPS) mediate immunomodulation, further study of the therapeutic opportunities of other immunomodulatory components and their potential risks is needed.

##### Breast Milk-Derived Extracellular Vesicles (BM-EVs) 

BM-EVs are characterized by their large numbers, high diversity [345], and enrichment of immune-related miRNAs capable of conferring immunomodulatory effects to the infant [346]. Recently, various studies have shown that BM-EVs have direct pharmacological effects, including significant anti-inflammatory, tolerogenic, and anti-apoptotic effects. Porcine BM-EVs promoted cell proliferation and reduced cell apoptosis by decreasing deoxynivalenol-induced injury via upregulation of miRNAs in the p53 pathway in vitro [347], as well as preventing LPS-induced injury via downregulation of inflammatory cytokines [348]. Human BM-EVs had a potency to protect against necrotizing enterocolitis by increasing cellular proliferation and decreasing apoptosis in vitro [349]. Interestingly, bovine BM-EVs attenuated colitis by upregulating the inflammatory protein A20 in the NF-κβ pathway and downregulating colitis-associated miRNAs in vitro [350]. Furthermore, bovine BM-EVs protected against cisplatin-induced toxicity in vitro by increasing macrophage proliferation and expression of β-catenin, p21, and p53 [351]. Additionally, BM-EVs diminished arthritis via improving cartilage pathology and bone marrow inflammation [352]. The same EVs were proven effective against breast cancer via promoting apoptosis and reducing oxidative stress and inflammation markers in vitro and in vivo [353]. Regardless of the source, BM-EVs in general can have potent anti-inflammatory, immunomodulatory, and anti-apoptotic effects, which can be used to treat various inflammatory disorders. 

### 3.2. Pharmacokinetics of Extracellular Vesicles

The concept that EVs are conveyors of information and functionality enhances their application as drug delivery platforms; thus, it is essential to understand the pharmacokinetics of EVs. This section addresses issues concerning machinery involved in the biogenesis of intrinsic EVs (release and uptake), as well as the biokinetics of extrinsic EVs, including absorption, distribution, metabolism, and excretion (ADME).

#### 3.2.1. Pharmacokinetics of Intrinsic Extracellular Vesicles 

Endocytosis of molecular cargo and early endosome formation is the first step in EV biogenesis [354,355]. Cargo sorting within the early endosome involves three pathways. Cargo that needs to be recycled will be placed in the peripheral tubular domains of endosomes that will separate to integrate into the Golgi network or the plasma membrane. These cargoes fused to the plasma membrane are either conveyed directly into pinched microvesicles or incorporated into released exosomes. Cargo not destined for recycling will concentrate in the central endosomal region and maturate to form late endosomes. These late endosomes either fuse into lysosomes and then degrade or fuse into the plasma membrane and are subsequently released as exosomes [354]. In contrast to changes in subcellular localization, the maturation of endosomes is accompanied by changes in their membrane. Changes in membrane composition allow downstream mobility and sorting, such as ceramides, instead of sphingomyelin, and Rab11, essential for trafficking in late endosomes, instead of Rab5 in early endosomes [356,357]. Endosomal vesicular maturation of certain membranous regions initiates engulfment and budding away from the cytoplasm to generate ILVs that enclose the cargo into late multivesicular endosomes, i.e., MVBs [3]. MVBs that have fused into lysosomes lead to the degradation of ILV cargo, whereas MVBs that have fused into plasma membrane lead to the secretion of ILVs into the extracellular space as exosomes. 

##### Extracellular Vesicle Release 

After MVBs are delivered to the plasma membrane, they undergo docking and fusion with the cell membrane via key players such as v-SNAREs (on vesicles), t-SNAREs (on target membranes), Rab GTPases, tethers, and additional proteins [358]. Complexes of one v-SNARE molecule and three t-SNAREs molecules occur between the fused membranes. Rabs include Rab27a, Rab27b, and Rab35, which recruit tethers for the binding of SNARE proteins (i.e., v-SNAREs and t-SNAREs) and vesicular (i.e., EVs) docking at the cell membrane [358,359,360]. SNARE proteins, such as VAMPs (v-SNAREs), syntaxins (t-SNAREs), and SNAPs (t-SNAREs), play a role in facilitating the fusion and hence secretion of EVs. For example, VAMP7 localized to late endosomes forms VAMP7-syntaxin1-SNAP25 and VAMP7-syntaxin3-SNAP23 complexes, promoting fusion [361]. In an Alzheimer’s disease model, tau-bearing vesicles fuse to the cellular membrane of neurons through late endosomal v-SNARE VAMP8 participation [362]. Hepatitis C virus-carrying MVBs fuse to the cellular membrane via syntaxin4 of infected cells and hence spread infection via released EVs [363,364]. In models of Parkinson’s disease, the correlation between increased α-syn concentration and decreased interaction between syntaxin4 and VAMP2 results in decreased EV secretion [365]. In prostate cancer cells, knockdown of t-SNARE (syntaxin 6) significantly decreased EV production and thus reduced drug resistance conferred by this secretion [366]. Although most SNARE proteins are cell type-specific, it is clear that VAMP7 and SNAP23 are ubiquitous hubs of the membrane fusion process [361]. Post-translational modifications of SNARE proteins, such as decreased SNAP23 O-GlcNAcylation, enhances its interaction with syntaxin4 and VAMP8 leads to increased secretion of EVs, whereas a similar effect is produced by phosphorylation of SNAP23 and H1 receptor activation in Hela cells [367,368]. In addition, a study in cancer cells showed that PKM2 involved in the phosphorylation of SNAP23 at Ser95 upregulates the secretion of EVs [369]. In contrast to phosphorylation, RNAs have been employed in the regulation of SNARE and the secretion of EVs. For example, in models of non-small cell lung cancer, miRNAs (134, and 135b) inhibit the SNARE protein YKT6 and reduce the secretion of EVs [334]. Similarly, in pancreatic cancer cells, long non-coding RNA (lncRNA) PVT-1 and HOTAIR regulates YKT6 and VAMP3 [370] and SNAP23 with VAMP3 colocalization [371], respectively, thereby playing a role in the fusion of MVBs with the plasma membrane. 

##### Extracellular Vesicle Uptake 

Generally, fusion of MVB with the cell membrane is followed by secretion of ILVs into the extracellular space as EVs (i.e., exosomes). Mechanisms and players of EV targeting are still unknown, and the question of how many EVs will be delivered randomly rather than specifically remains unanswered [372]. There are three pathways through which EVs interact with target cells: (a) direct interaction between cell membrane receptors and EV surface proteins; (b) cell–EV membrane fusion; and (c) endocytosis [359,373,374,375,376]. Moreover, to date, EV surface molecules such tetraspanins, immunoglobulins, proteoglycans, and lectin receptors are implicated in EV target cell binding through unknown mechanisms [359,377,378]. The most therapeutically important EV ligands that have receptors on cancer cell surfaces are PD-L1, TNF, FasL, and TRAIL, and they are considered potential anti-cancer targets. The most efficient pathway for intracellular delivery of EV cargo is through EV–cell membrane direct fusion. Indeed, such a mechanism does not always occur, as evidence indicates that the dominant mechanism for uptake of EVs by the cell is endocytosis, where intact EVs are engulfed, bound by the plasma membrane, and eventually joined to endosomes [372]. This would exacerbate the dilemma of intracellular delivery, given the needs of EV cargo to escape from endosomes into the cytoplasm, i.e., “endosomal escape”, avoiding lysosomal degradation, cellular recycling, or exile into the extracellular space [379,380,381]. There are a few proposed mechanisms based on pH-dependent permeability for endosomal escape of cargo into the cytoplasm, where it can carry out its specific function [381,382]. One of the major hurdles in utilizing EVs for clinical application is the understanding of the mechanisms of EV cargo release into the cytoplasm and the poor predictability of the process across different cell types [381,383,384]. Similarly, much remains to be understood about the transmission of EVs in the blood and the crossing of the endothelial layers of blood vessels. However, recent studies on the passage of EVs across the blood–brain barrier (BBB) have indicated that transcytosis is the most widely accepted mechanism for transporting EVs across the endothelium [385]. Breast-cancer derived EVs are taken up by endothelial cells via clathrin-mediated endocytosis, sorted by Rab11 for exocytosis at the basolateral membrane, and finally secreted from the cell through interactions between EVs v-SNARE and VAMP-3 and membrane-associated t-SNAREs SNAP23 and syntaxin 4 [386]. Although it is not clear whether endothelial cells are involved in the internalization or attachment of EVs, surface heparan sulfate proteoglycans have been shown to be involved in cellular endocytosis of EVs [387]. Another mechanism that facilitates this is EVs’ adsorptive transcytosis through interactions between positive and negative molecular charges [388]. 

#### 3.2.2. Pharmacokinetics of Extrinsic Extracellular Vesicles

Compared to many synthetic drug delivery systems, the exceptional EVs that are well-tolerated in vivo as mediators of intercellular communication are beginning to highlight their usefulness as effective drug delivery platforms for a range of therapeutic macromolecules. These advances and their applications can be made possible by technological advances in the labeling and understanding of the in vivo pharmacokinetics of exogenously administered EVs. EVs have the unique attributes of stability in circulation, biocompatibility, immune tolerance, and the ability to cross all biological barriers, entering all organs, including the central nervous system [389,390]. Although therapeutic EV research has evolved from in vitro studies to pre-clinical models to clinical trials [389], successful clinical translation has various obstacles. This section will focus on labeling and engineering EVs as tools to study their in vivo kinetics and potential for drug delivery and targeting.

##### Extracellular Vesicle Labelling 

In general, labelling of EVs can be performed in two ways, either by general labelling of EV-associated macromolecules or by labelling of an EV-associated specific macromolecule [391]. The bond established between the lipophilic functional groups of the fluorophore-conjugated dye and the EV lipid bilayer will be non-covalent. Various lipophilic tracer dyes, such as PKH67 and DiR/DiL/DiD, cover a wide range of emission wavelengths for better in vivo penetration through tissues [392,393,394]. Although these dyes are capable of rapid and efficient labeling of EVs without altering the EV-producing cells, they tend to aggregate into micelles similar in size to EVs and also potentially label non-EV particles [395]. In addition, the non-covalent bond promotes a high risk of transferring the EV-bound dye to the plasma membrane, as well as altering the properties of EVs, thereby affecting the biodistribution of EVs in vivo [394,396]. In contrast to the non-covalent anchoring of lipids, the fluorophore NHS ester covalently binds to the surface protein amine groups of EVs [397,398]. These covalent conjugations can alter the surface proteome of EVs, thereby affecting their interactions with other proteins. Furthermore, these dyes can label non-vesicular proteins, thus making them inaccurate. Nevertheless, dyes/tracers that are currently being produced are very stable, with a half-life of a few days to weeks [399,400]. Apart from fluorescent dyes, EVs can be labelled with various radiotracers, such as ^99m^Tc-HMPAO [401], ^125^I-IBB [402], and ^111^Indium-oxine [403]. Superparamagnetic iron oxide nanoparticle (SPION)-loaded EVs can be employed for biodistribution studies utilizing magnetic resonance imaging (MRI) [396,404]. Despite the high sensitivity in vivo of radiolabeling and MRI, the high infrastructure cost makes it difficult to implement in basic science research. In contrast to exogenous EV labelling, genetically engineered cells can generate fluorescent or bioluminescent protein-labeled EVs [396,398,402,405,406,407]. To label a specific population, genetically engineered producer cells express a reporter protein fused to the EV sorting domain to allow loading of the reporter protein during EV biogenesis. Thus, the CD63 and eGFP fusion protein can promote eGFP sorting in 30–40% of EVs; therefore, each carries 30–60 eGFP molecules on average [408]. This approach can similarly be exploited to label other EV sorting domains, such as CD9, CD81, syntenin, and Gag [408,409]. However, not all EV protein domains, such as ALIX, SIMPLE, and syndecan, can be engineered and characterized because the efficiency is relatively low [408]. Generally, genetic engineering approaches provide an effective way to tag a specific group of EVs, either with fluorescent proteins, e.g., GFP, RFP, etc., or bioluminescent proteins, e.g., Gaussia-, Firefly-, and Nano-luciferase. Disadvantages of these approaches include failure to label all EVs and the requirement for genetic engineering of the produced cells, which is challenging for some cell sources. Furthermore, overexpression of specific EV-sorting proteins may change EV biogenesis and/or proteome, thereby changing the biodistribution of EVs. Because there is no perfect EV reporter or labeling method, and because each method has a certain degree of advantages and disadvantages, the choice of labeling method should be based on indication and feasibility.

##### Extracellular Vesicle Engineering 

To neutralize the problem of EV clearance and to promote additional hepatic delivery of EVs, endogenous and exogenous EV surface engineering strategies have been utilized. CD47 surface protein, a potent “do not eat me” signal, has been shown to be expressed in many normal and tumor cells. Namely, CD47-expressing TC-EVs can inhibit phagocytosis by interacting with SIRPα on macrophages, by which tumors can evade the innate immune system. Previously, there have been several delivery vectors, such as lentiviruses engineered with CD47 to inhibit phagocytosis and liver clearance [410]. Similarly, EVs engineered with CD47 inhibit their uptake by monocytes and prolong their half-life in circulation [411]. Similar observations have been made in other studies that have utilized CD47 overexpression [412,413], as well as expression of CD47-resembling molecules such as CD55 and CD59 on the surface of EVs [330]. The relatively long in vivo half-life of 3 weeks of albumin, the most abundant human plasma protein, has brought much interest for its use in drug delivery for a range of biotherapeutics, either by direct incorporation or via a binding domain on the surface of the carrier [414]. Similarly, insertion of albumin-binding peptides into the extracellular loop of CD63 prolongs the circulation half-life of EVs [415]. The goals behind this EV engineering are to improve extrahepatic delivery and to extend the plasma half-life of EVs. A very common strategy in pharmaceutics to improve therapeutic pharmacokinetic properties is the hydrophilic coupling of polyethylene glycols (PEGs, PEGylation) to prevent the electrostatic interaction between plasma proteins and the delivery carrier [416]. Lipid nanoparticles (LNPs) and LPs are the most clinically validated delivery systems utilizing this strategy [417]. Similarly, the use of this strategy in EV research will result to an extension of the plasma half-life, a decrease in hepatic uptake, and an increase in extrahepatic delivery [418].

## 4. Pharmaceutical Applications of Extracellular Vesicles

**Figure 2:**
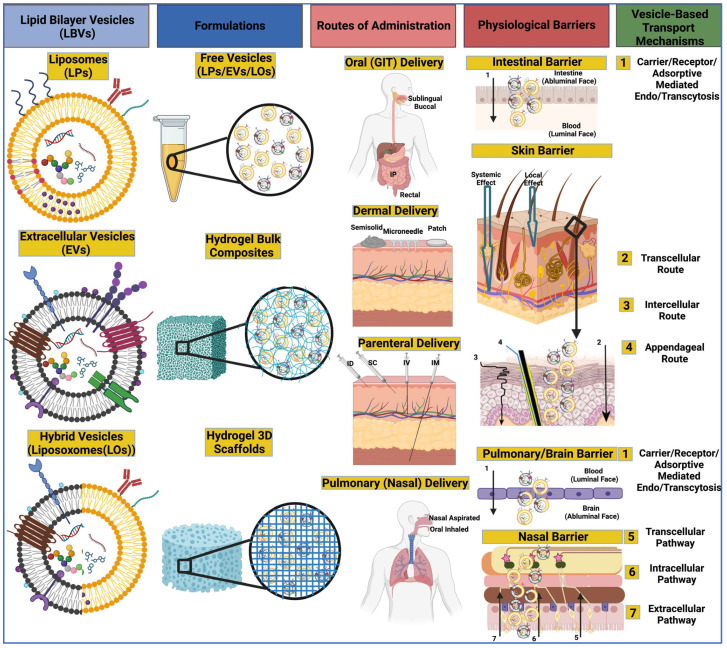
Challenges of administration of lipid bilayer vesicles (LBVs: LPs, EVs, and LOs) in vivo. The formulation of LBVs ranges from free suspensions to bulk composites and 3D scaffolds within functional biomaterials. The route of administration determines the most appropriate LBV formulation to use to achieve the intended effect. By adjusting the composition and administration of LBVs, it is possible to facilitate their delivery across normal physiological barriers. Epithelial and endothelial cells lining these barriers internalize LBVs by different mechanisms (created with https://app.biorender.com/ (accessed on 25 December 2023)).

In contrast to LPs that have been used clinically as well-established nanocarriers for drugs in the treatment of various diseases [419], the development of EVs [420] as well as hybrid liposoxomes (LOs) [421] is still in the pre-clinical stage. One of the hurdles with EV-based therapies is designing them as suitable dosage forms for specific applications. With the wide applications of EVs in the management of various diseases, delivery strategies can be critical to achieve the optimal therapeutic effect. Although intravenous (IV) injection is the most common method of administering EVs, other drug delivery routes may be considered, including oral, nasal, or pulmonal, depending on the application of EVs (Figure 2). This section will closely look at several decades of designing LP formulation dosage forms for various routes of administration, establishing dosing regimens, and in vitro–in vivo translational studies, as well as pharmacokinetic evaluations that may help pave the way for the formulation of better EV-based dosage forms. 

### 4.1. Applications of Lipid Bilayer Vesicles for Drug Delivery

Compared to several NP delivery systems, lipid bilayer vesicles (LBVs) are versatile platforms for drug packaging and delivery. Based on their origin, they are classified into synthetically originated LPs, biologically originated EVs, as well as hybrid LOs originating from the fusion of LPs and EVs [253]. LPs are self-assembled synthetic NPs that provide a prominent platform consisting of fatty acids and lipids centered in a spherical bilayer membrane surrounding an aqueous chamber [422] in which both hydrophilic and hydrophobic molecules can be encapsulated. LPs improve the pharmacokinetics of the incorporated molecules by increasing their circulation time and overcoming barriers, such as the BBB [423]. LPs were classified based on structure into unilamellar, multilamellar, and multivesicular constructs. The LP preparation method determines the structure and size of the LPs produced, which in turn determines the encapsulation capacity and the drug release [424]. The membrane fluidity of LPs associated with their composition has also been the basis for their classification into either non-flexible (non-deformable, classical, and conventional) or flexible (deformable and elastic) LPs. The charge of the LPs’ phospholipid head groups depends on the characteristics of the surrounding environment, including pH, temperature, and ionic strength. Thus, LPs’ ζ-potential is a key parameter affecting the stability of liposomal dispersions and plays a role in the interaction between LPs and the biological environment [425,426]. Studying these interactions is crucial for predicting the biological fate of LPs, including corona formation and adsorption onto the cell membrane [427]. 

In the context of drug delivery, all advantages of LPs reported in the literature also apply to EVs. EVs have revealed great potential for integrating many small molecules [428], proteins [429], nucleic acids [430], and theranostics [431] to be loaded and transported via EVs for therapeutic and diagnostic applications. Moreover, EVs in a hybrid platform [432,433] incorporate other nanovesicles [434,435] that provide them with superior biomimetic or drug loading purposes. In contrast, diverse combinations of LPs with chemical and biological entities improve their physicochemical properties and stability, which in turn enable controlled drug release and optimize their interactions with the biological environment [436]. These combination approaches include modification with polymers [437], peptides and proteins [438], and nucleic acids [439], as well as coating with [440] or encapsulating other [441] nano-entities and hybridizing with the cell membrane [442] or EVs [443]. Apart from these approaches, intact EVs are more complex due to their biological origin, and thus meet the complexity requirements of the optimal biological level of nanomedicine. This ideality is due to the hundreds of different types of lipids, proteins, and carbohydrates as well as internal cargoes and surface-bound molecules [253]. In addition, further design of EVs can be accomplished using EV parent cells engineering [444,445,446]. Although very simple LP systems can be produced on a large scale, EVs may offer the possibility to design more complex membrane nanovesicles. EVs outperform LPs through their remarkable similarity to the cell membrane and are thus more biocompatible and safer [447]. Employing patient-derived EVs makes it a very promising tool in the context of personalized medicine [448]. From a pharmacokinetic point of view, EVs, compared to LPs, have superb circulation time [393,449,450] in crossing biological barriers and exert physiological, pathological, and therapeutic effects [388,451]. In order to bridge the bench-to-market gap in the clinical translation of both LBV (LP and EV) drug delivery products, a range of hurdles must be overcome. These barriers include (1) fully disclosing the physicochemical properties of the interaction of the lipid bilayer with the biological environment [452], (2) employing smart strategies to control drug release and concentration at the site of action [453], (3) advanced production techniques with the highest levels of particle homogeneity, drug content uniformity, and batch reproducibility, scalability, and sterility [454], (4) preserving storage stability through innovative formulations [427], and (5) ensuring clinical trial success by fitting in silico, in vitro, and in vivo models to provide the highest simulation of the PK-PD of the human body in in vivo studies [253,455]. The following parts will deal with LP-based dosage forms for various routes of administration and how they may pave the way for better EV-based dosage forms.

#### 4.1.1. Oral Delivery

Oral administration of LPs is hampered by their instability and difficulties in bypassing bio-membranes, as their initial application with insulin delivery [456] was neither reproducible nor predictable [457]. Attempts have been made to improve their oral delivery [458,459,460,461,462,463] by adding polymers or ligands to modify their lipid compositions, which can enhance their stability and permeation. In parallel, naturally produced EVs have recently gained much research interest as a platform for miRNA and drug delivery. The speculation that lipids support the intestinal transmission of miRNAs [464] has opened up another research direction, namely studies on EV-based transmission and function after oral delivery both locally and systemically. The fact that most water and nutrient absorption takes place in the gut could also be true for orally delivered EVs [465]. The digestion stability of bovine milk EVs containing miRNAs was evaluated in vitro by subsequent incubation in three solutions simulating oral, gastric, and intestinal phases of digestion, respectively. It has been shown that about 50% of all miRNAs survive the oral and gastric phases of digestion. Moreover, in in vivo oral administration in mice, EVs were detected in various distant tissues [466]. These results provide indirect evidence of the digestive stability of EVs, allowing them to reach the intestine after oral administration. Furthermore, after absorption through the intestine [467], therapeutic EVs can exert predictable effects at distant sites. The present observations suggest the involvement of the “neonatal” Fc receptor in the uptake of intact EVs [468] and the role of integrins in both tissue trafficking [469] and subsequent EV uptake by cells [470]. Attempts to evaluate orally administered fluorochrome-labeled EV bioavailability and tissue biodistribution in mice after oral gavage have demonstrated the presence of vesicles in the intestine, liver, spleen, kidney, lung, heart, and brain [471]. However, these in vivo attempts have failed to estimate the precise efficacy of EVs’ passage through the GIT, [472] because it relied on the detection of EV-containing miRNAs rather than the vesicles themselves [473]. Oral drug administration is the preferred route for clinicians and patients. EVs’ superiority to LPs in oral delivery is due to features including fast internalization, low immunogenicity even at repeated doses, physiological stability, and feasibility of modification of internal and surface components, which generates specific and controlled release of internalized or loaded therapeutic molecules. In general, nanocarriers (including LPs and EVs) are a colloidal delivery system for drugs with a particle size of less than 500 nm [474]. Different types of nanocarriers cross the intestinal epithelium using different mechanisms [475]. One such mechanism is paracellular transport, which covers the diffusion of particles between 0.5 and 20 nm across the intestinal epithelial barrier and is therefore impractical due to the limited physical dimensions between cells [476,477]. Conversely, disruption of the intestinal barrier, either due to inflammatory diseases or treatments that reduce the tightness of the epithelial barrier, allows the passage of larger EVs over 200 nm [478]. The other mechanism is transcellular transport, mainly via endocytosis by epithelial cells and phagocytosis by M cells, where the former accounts for 90–95% and the latter 1% of the total cells of the GIT [475]. Wu et al., 2022 found insulin-loaded bovine milk EVs showing efficient internalization through multiple active endocytic pathways into the epithelium [479]. The authors, as well as Betker et al., 2019, suggested that since milk is a nutrient, milk EV uptake is mediated by peptides, amino acids, glucose transporters, and the neonatal Fc receptor (FcRn) [468,479]. In contrast to several studies demonstrating a rapid clearance rate of circulating exogenous EVs after IV injection (~2–30 min) mediated by the reticuloendothelial system (RES), mainly macrophages [480], Munagala et al., 2016 found that bovine milk EVs remained circulating for at least 24 h after oral administration in nude mice [481]. The same group tested milk EVs for oral paclitaxel (PTX) administration in a lung tumor xenograft model, demonstrating that orally administered PTX-EVs significantly inhibited tumor growth compared to the same dose administering PTX intraperitoneally. These PTX-EVs showed significantly less systemic and immunologic toxicities compared to IV PTX [482]. Soo Kim et al., 2016 showed that murine RAW 264.7 macrophage-derived EVs loaded with PTX are more than 50-fold cytotoxic to drug-resistant MDCK_MDR1_ (P_gp+_) cells in vitro [483]. 

To understand the true clinical potential of oral administration of EVs, the question that remains to be answered is why EVs absorbed from the GIT have a longer circulating half-life than observed in systemically injected EVs. Bardonnet et al., 2006 suggested that NP size is necessary for gastric retention, as particles < 7 mm are evacuated efficiently [484]. Thus, the size range of EVs of 50–200 nm [5] is unlikely to have any biological effect in the stomach due to poor gastric retention. However, modifying EVs with mucoadhesion strategies using polymers or phospholipids in their surface membrane could give them time to induce the desired GIT biological changes [475] as well as drug delivery. In accordance with bovine milk EVs, the addition of casein has been shown to enhance the uptake of EVs derived from human cardiosphere stromal cells. Modification of EVs with casein also presented an increased biological effect compared to unmodified EVs in cardiac dysfunction [485]. Munagala et al., 2016 showed that the addition of folic acid to the surface of bovine milk EVs loaded with withaferin A resulted in a reduction in tumor size in a murine model of lung cancer. This response was attributed to folic acid, which either enhanced the stability of EVs in the GIT or targeted cancer cells after systemic circulation was reached [481]. These data indirectly support bovine milk EVs as nanocarriers for oral drug delivery. Similar to PEGylated LPs, Warren et al., 2021 modified the surface of milk EVs with PEG, thereby decreasing hydrophobic interactions with mucin lining the intestinal lumen, increasing uptake by epithelial cells, and delivering siRNA loaded in vitro [486]. Although oral delivery of EVs offers various physiological and practical advantages compared to other routes, there is still a need for further investigation into their safety, stability, pharmacokinetics, and biodistribution features before they can be widely used as drug vehicles or nutritional supplements. 

#### 4.1.2. Dermal Delivery 

Exogenous molecules can cross the skin by transcellular permeation, paracellular transport, and absorption via skin appendages, including hair follicles and glands [487]. LPs and EVs have tremendous potential to deliver active pharmaceutical ingredients (APIs) to skin structures [488,489]. Some studies have shown that intact LPs permeate the stratum corneum (SC), the outermost layer of the epidermis, after topical application [490]. The composition of LPs [491], size, ζ-potential, and membrane fluidity and elasticity play an important role in the rate and depth of skin penetration. A higher proportion of studies reported hydrogel formulations of LPs compared to other semisolid dosage forms, including ointments and creams for topical applications. Ex vivo models of human skin have been used to study the time-dependent penetration of stem cell-derived EVs through the SC and their internalization by dermal fibroblasts [492]. Zhang et al., 2021 reported topical application of an aqueous dispersion cream (oil-in-water emulsion) of MSC-EVs on explanted human skin cultures, resulting in less than 1% of the particles penetrating beyond the SC [493]. Furthermore, in vivo experiments in rat models involved very limited infiltration of MSC-EVs into the SC when administered topically [494]. However, OMVs of the skin pathogens *Staphylococcus aureus* (*S. aureus*) [495] and *Malassezia furfur* [496] may penetrate deeper layers of the skin, especially when the SC is removed or damaged. Furthermore, engineering *Escherichia coli* (*E. coli*) OMVs with integrin-targeting peptides, RGP, resulted in excellent infiltration across epidermal barriers, mainly via the skin appendages and intracellular pathway, resulting in OMVs being widely present in the dermis [497,498]. Among many advanced formulation-based strategies, hydrogels stand out for their versatility and attractive properties as suitable dermatological dosage forms of LPs and EVs. Hydrogel-forming polysaccharides such as chitosan, alginate, and hyaluronic acid are a class of hydrogel biomaterials that are widely used in the food and pharmaceutical industry due to their abundance in nature, biodegradability, and biocompatibility.

#### 4.1.3. Parenteral Delivery 

The development of new drug molecules for the alleviation and treatment of various diseases is an ongoing and continuous process. However, at present, most of the developed new chemical entities have poor aqueous solubility and many undesirable physicochemical properties such as short half-life, extensive degradation, high protein binding, first-pass metabolism, and poor intestinal permeability [499]. Novel formulations are being developed for parenteral applications, which has improved PD-PK behavior of the drug with lower dosing frequency and minimal adverse effects [500]. Many APIs, especially small molecules, are not suitable for encapsulation in LP formulations intended for parenteral administration due to their inherent permeability and lipophilicity (partition coefficient). An API with a high permeability leads to premature leakage of vesicles, while too low a permeability means an inability to cross biological barriers. Solutions to such a problem include modifying lipid composition of LPs, adding a permeation enhancer, or modifying the chemical structure of the API as a prodrug. Balouch et al., 2023 reported that with modification of the chemical structure of the four parental drugs (abiraterone, cytarabine, 5-fluorouracil, and paliperidone), both permeability and lipophilicity could be systematically converted to the desired LP formulability window [501]. In contrast, EVs, as novel drug delivery platforms with amphiphilic loading capacity, offer several advantages that overcome many limitations imposed by conventional and advanced LP parenteral nanoformulations. Among these, the factor that plays a role in the usefulness of EVs for systemic administration is the surface protein CD47, which limits the uptake of EVs by macrophages, thus prolonging the circulatory half-life of exogenously administered EVs. The same benefit has been reported for synthetic NPs such as LPs decorated with CD47-derived peptides [502]. Selection of a pharmaceutical nanocarrier requires the fulfillment of two basic principles: protecting the contained drugs from inactivation in the in vivo environment and releasing the contained drugs without inducing an immune response to the nanocarriers. In this sense, the employment of EVs in drug delivery is superior to existing nanocarriers like LP- and polymer-based nanocarriers. This superiority is based on several features [389]. EVs arise naturally from normal cells, where their inclusions can transfer and alter the function of recipient cells. Compared with LPs, EVs can effectively attract nucleic acids, i.e., hydrophilic, and greatly improve the packaging efficiency. The ability of surface molecules of EVs to evade interaction with opsonin, antibodies, and coagulation factors helps avoid immune responses in vivo. Compared with LP- and polymer-based nanocarriers, EVs have higher stability in body fluids such as blood. Finally, EVs derived from special cells including MSCs or immature DCs as well as EVs derived through native or engineered molecules present on the surface can have a targeting effect by selectively binding to recipient cells [389]. Although cargo-loaded EVs are futuristic multifunctional nanotherapeutics, the combination of LPs and EVs (i.e., hybrid liposoxomes (LOs)) promotes drug delivery systems, as each system contributes to improved stability, drug loading capacity, and drug release controllability [503]. Among the various avenues of parenteral administration (IV, intramuscular, intradermal, subcutaneous, intraperitoneal, intra-articular, intrathecal, intratumoral, etc.), to date, IV administration of EVs as therapeutic agents or carriers prevails in treatment strategies for various diseases [504]. However, there will be a future trend to integrate EVs with smart technologies to achieve real-time detection and control of drug release as well as personalized drug therapy and precision medicine. 

#### 4.1.4. Pulmonal Delivery 

Inhalation therapy offers an attractive and noninvasive method of drug delivery for local and systemic treatments. By directly inhaling drugs, pulmonary bioavailability can be improved, while subsequent adverse effects can be reduced [505,506]. However, drug formulation and aerosol deposition are critical obstacles that hinder therapeutic efficacy. Nanomaterials provide a solution by altering the drug’s size, solubility and surface chemistry to become compatible with the pulmonary microenvironment [506,507,508]. Developing uniform, loadable NPs for a range of pulmonary therapeutic applications and determining their distribution characteristics upon inhalation would clarify cellular targeting and optimize drug dosage. mRNA-loaded lipid NPs have demonstrated therapeutic efficacy in eliciting systemic immunity against severe acute respiratory syndrome coronavirus 2 (SARS-CoV-2) infection as a vaccine for intramuscular (IM) injection [509,510,511,512], opening the application of mRNA-based therapeutics for the treatment of other lung diseases across different inhalation devices, such as nebulizers [513,514]. However, extensive formulation of lipid NPs is needed to improve mRNA translation and pulmonary bioavailability for inhaled delivery. Therefore, EVs of natural origin provide bio-alternatives to synthetic lipid NPs that are naturally optimized for mRNA encapsulation and cellular delivery [515]. Recently, Wang et al., 2022 successfully designed an inhalable vaccine using recombinant SARS-CoV-2 receptor-binding domain (RBD) conjugated to lung-derived EVs (lung EVs) to elicit local lung immunity against SARS-CoV-2 infection. In addition, its stability at room temperature for three months outperforms the requirement of mRNA-loaded lipid NP vaccines for cold chain transportation [516]. The use of lung EVs as nanovesicles for inhaled drug delivery may increase drug retention and efficacy by more efficiently avoiding immune clearance and targeting pneumocytes. Besides drug delivery, lung EVs themselves have demonstrated therapeutic benefits. In a rodent model of idiopathic pulmonary fibrosis, lung EVs better restore lung function and reduce the severity of fibrosis compared to their MSC-EV counterpart [517]. Furthermore, inhaled EV therapeutics are superior and outperform LPs, as they are naturally optimized to distribute and retain mRNA and protein cargo components to the lung after inhaled delivery [518]. 

Targeting EVs to a specific organ presents the challenge of rapid clearance after systemic administration, mainly via the liver. A study to minimize liver clearance was performed by Cober et al., 2023, which is based on the ability of porous microgels to engraft and increase the survival of transplanted cells. They encapsulated EVs and showed that lung targeting was improved, thanks to EVs’ size-based retention within the pulmonary microcirculation [519]. The existence of lung EVs in human airway mucus and their less obstructed movement facilitates crosstalk between lung-resident parenchymal cells and/or immune cells. This concession was used by Kwak et al., 2023, who demonstrated that Adeno-associated virus serotype 6 (AAV6) associated with EVs and secreted from vector-producing HEK-293 cells was a safe and effective platform for inhaled gene delivery. In contrast, standard preparations of AAV6 alone as well as physical mixtures of individually prepared EVs and AAV6 failed to mediate EV-AAV6 interaction or to improve gene transfer efficacy [520]. EVs, as cell-free therapeutics naturally loaded with various bioactive molecules, offer several advantages for clinical respiratory applications. First, small-sized EVs facilitate their inhalation and deposit within the small bronchioles and alveoli. Second, the lipid bilayer structure of EVs grants them stability in tissues and body fluids. Third, EVs show lower levels of immunogenicity and toxicity compared to cell therapies. 

#### 4.1.5. Local Delivery 

Local EV administration is beneficial for delivering EVs to well-defined lesions, thereby limiting systemic circulation [521]. This type of EV application ranges from topical administration to more complex radiological, ultrasound-, or endoscopy-guided routes. For instance, EVs embedded in hydrogels facilitate their delivery and retention at the site of action while providing a combined mechanical effect [522]. Although orally administered EVs were distributed to the liver, lung, spleen, ovary, colon, kidney, pancreas, and finally the brain four days after administration, IV-administered EVs accumulate mostly in the liver [481]. The ability of EVs to cross the BBB bidirectionally [385,523] makes EVs attractive as nano-biocarriers for drug delivery to the brain. Betzer et al., 2017 found that intranasal administration of the EVs resulted in a significant degree of enrichment of EVs in the brain [524]. Similarly, Han et al., 2022 devised an inhalation nebulizer for EVs and found that they are almost exclusively enriched in the lungs and not in other non-target organs, within 7 days [525]. Local application of EVs either by injection or direct coverage of the trauma site reduces their clearance by circulation and enrichment in non-target organs. However, due to the complexity of the trauma environment, EVs easily degraded and become inactive [526]. In order to evade the premature clearing and maintain the desired therapeutic effect over time, biodegradable, sparse, and porous hydrogels can be employed to carry EVs [527,528]. Wang et al., 2022 evaluated the bio-removal rates of EVs applied directly to local wounds versus those loaded with hydrogel. It was found that the former was almost completely removed within four days, whereas the latter was uniformly retained on the fourth day [529]. Similarly, Kwak et al., 2022 found that PEG-based hydrogels loaded with EVs for wound application barely reached the liver or kidneys and mainly acted on the skin [530]. To achieve a more localized and targeted delivery of EVs, hydrogels can be injected locally into the target organ or prepared as microneedle patches for topical application [528]. In contrast, the challenges of delivering hydrophobic chemotherapies require the development of a drug delivery system that targets tumor sites. Thus, EVs loaded with anticancer drugs can improve their solubility and reduce toxicity, while the use of ligands grafted onto the surface of engineered EVs can improve their targeting and efficacy [531]. Similar to chemotherapies’ poor pharmacokinetics, the susceptibility of current mRNA therapeutics (Pfizer–BioNTech’s mRNA-BNT162b2 and Moderna’s mRNA-1273 COVID-19 vaccines) to degradation [532] increases the need for an effective delivery system. Although lipid NPs could efficiently deliver mRNA intracellularly, a portion of the internalized mRNA continued to function through EV secretion, containing more molecules with similar biological functions. Thus, EVs can be considered a functional expansion of lipid NPs [533] and are best used to protect mRNAs as their loading vehicle. 

### 4.2. Applications of Hydrogel Platforms for Lipid Bilayer Vesicle Delivery 

Hydrogels are three-dimensional reticulation structures based on cross-linked hydrophilic polymers with excellent ability to absorb and retain water and biological fluids [534]. They can be classified based on several characteristics, including source (natural vs. synthetic), chemistry (polysaccharide, peptide/protein, miscellaneous; homo- vs. co-polymer), charge (neutral, cationic, anionic), cross-linking mechanism (physical vs. chemical), and biodegradation. Due to their biological tissue similarity, drug and NP loading capacity, and sustained release property, hydrogels have been extensively used in drug delivery and tissue engineering [535]. 

Approaches for loading LBV (prepared LPs, extracted EVs, or LOs)-based hydrogel platforms include the following: (i) *breathing method*—beyond removing excess water from the swollen hydrogel with a solvent, the exposed voids will be occupied by LBVs to obtain LBV-loaded hydrogel platforms [536]; (ii) *mix and crosslink*—by directly mixing LBVs with the hydrogel precursor solution followed by the addition of a crosslinking agent or by a physical crosslinking method [537]; and (iii) *in situ gel formation*—by mixing LBVs and polymers and injecting them with a crosslinking agent into the target site using a double-lumen syringe [538]. Biocompatibility and structural porosity allow hydrogels to act as carriers, prolonging the retention time of LBVs at the site of action and slowing their release [539,540]. Adjusting the swelling rate, surface charge, and degradation rate are all methods to tune the porosity of hydrogels, thereby tuning the loading and release of LBVs [541,542]. In contrast, the LBV-related properties (particle size and lipid composition) dictate the membrane stiffness of LBVs as well as the interaction with the hydrogel matrix, which directly affects the release kinetics of LBVs from the hydrogel platform [543,544]. The diffusion phenomena mainly describe the release pattern of LBVs’ payload from hydrogels, which is directly controlled by the mesh size, swelling deformation, and degradation of the polymeric network [545]. In addition, internal factors such as temperature [546], pH and ionic strength [547], specific enzymes [548], and oxidative state [549], as well as external factors such as electromagnetic waves [550], ultrasound waves [551], electric current [552], and magnetic field [553], can be introduced at the research level to trigger the release of LBVs’ payload from hydrogels. These factors that affect the release of LBVs from hydrogel platforms also affect the release of cargo from those LBVs, thereby measuring the ratio of the amount of drug released in the form of intact LBVs to the total amount of drug released, i.e., free drug and drug incorporated into released LBVs separately [544]. Thus, more controllable drug delivery can be obtained through sustained and multi-step-release LBV–hydrogel composites. In this section, we will review some of the innovative platforms with a focus on LBV-based hydrogels.

#### 4.2.1. Wound Dressings

Hydrogels are an ideal alternative for skin and wound dressing, because of their ability to eliminate infection, absorb trauma exudate, maintain water balance and gas exchange, and enclose, protect, and deliver bioactive molecules [554]. Trauma dressings are used to protect damaged tissue from environmental contaminants and infections. Dressings effectively support the healing process by creating an ideal hermetic wound environment [555] characterized by its porous structure, viscoelasticity, and water content. Zhao et al., 2020 [556] incorporated human umbilical vein endothelial cell-derived EVs (HUVEC-EVs) into well-designed gelatin methacryloyl (GelMA) hydrogels, and completely dressed skin wounds with them. They demonstrated in vivo and in vitro that GelMA hydrogel dressings not only helped repair injured tissue, but also achieved prolonged release of loaded HUVEC-EVs.

#### 4.2.2. Microneedle Patches

In order to overcome the limitations of delivering conventional hydrogels to deep tissues across the skin barrier, a method involving the use of microneedles (MNs) has been implemented [557]. Common materials used in MN preparations include gelatin, polylactic acid–hydroxy acetic acid co-polymer (PLGA), polyvinyl alcohol (PVA), and chitosan, which have been used to deliver LBVs [558,559]. The soluble shell and core structural properties of MNs facilitate deep and sustained delivery of the bioactive payload, which synergistically promotes wound healing [558]. Yuan et al., 2022 prepared an MN patch comprising methacrylate gelatin/polyethylene glycol diacrylate (GelMA/PEGDA) hydrogel. Subsequently, the preloaded MN molds are subjected to optical or chemical crosslinking, followed by freeze-drying to obtain hydrogel MN-encapsulated HUVEC-EVs and tazarotene. After in vitro application, the active ingredients are released around the wound site; they promote collagen deposition, epithelial regeneration, and angiogenesis [559]. In contrast, Ma et al., 2022 designed a novel core–shell hyaluronic acid (HA) MN patch with ferrum-MSC-derived artificial nanovesicles (Fe-MSC-NVs) and polydopamine NPs (PDA NPs) encapsulated in the needle tips. The Fe-MSC-NVs loaded with cytokines are encapsulated in the inner HA core, whereas PDA NPs are encapsulated in the outer methacrylated HA (HAMA) shell of the MN tips [558]. Hierarchically, these procedures involve encapsulating LBVs into the shell/core of the needle tip and freeze-drying to complete the construction of MN composites. 

#### 4.2.3. Injectable Applications 

Injectable hydrogels can be applied not only to superficial wounds, but also to deeper tissues and organs [560]. The application of direct injection of hydrogels loaded with active ingredients, such as drugs, growth factors, and cells, into the damaged area allows for effective repair while reducing the need for tedious surgical procedures and hence the burden on patients. Thus, local injection of LBV-loaded hydrogels resulted in sustained local release of LBVs, which promoted repair and regeneration of injured tissues [560]. These results required hydrogels with shear thinning rheology (sol state) before injection and in situ gelation rheology (gel state) after injection via physiologically induced crosslinking [561]. Cao et al., 2021 [562] injected a hydrogel loaded with human urine stem cell-derived EVs (USC-EVs) intrathecally, which promoted angiogenesis and repair of spinal cord injury (SCI).

#### 4.2.4. Bioink-3D Bioprinting

Bioink-3D bioprinting is a method for creating hierarchically complex and customizable geometric shapes using computer-aided design software. Due to the excellent rheological properties of hydrogels, they can be used as bioinks in 3D printers for bioprinting scaffolds with tactile structure, porosity, and mechanical properties that can effectively load LBVs [563]. Born et al., 2022 [564] demonstrated that a 3D GelMA hydrogel loaded with MSC-EVs maintained their biological activity beyond 3D printing and photo-crosslinking. They also showed that the burst release of EVs could be reduced by optimizing the crosslinker concentration, while the porosity of the hydrogel and meshwork could be changed by altering the GelMA synthesis and crosslinking parameters, which in turn significantly affected the release of EVs. 

## 5. Conclusions

EVs play crucial roles in various biological processes and diverse cellular activities by mediating intercellular communications. Conversely, in pathological conditions, they contribute to the initiation, worsening, and resilience in various diseases. Understanding EVs’ regulatory mechanisms and function in different biological processes is deemed crucial for unlocking their clinical potential and applications. This, indeed, involves developing new therapeutic strategies or interventions based on regulatory mechanisms of EVs.

EVs derived from MSCs, specific tumor cells, dendritic cells, B lymphocytes, and macrophages hold significant therapeutic potential, without the necessity for specific manipulation, and they elicit anti-inflammatory, anti-apoptotic, pro-angiogenic, and proliferative effects. Additionally, engineering EV-producing cells will enhance their therapeutic potential, and this can be achieved through genetic engineering, changing cell culture conditions, and stimulating cells with factors such as drugs and cytokines. Specific examples, such as the regenerative effects of MSCs in the lungs and breast milk-derived EVs, with anti-inflammatory and immunomodulatory properties, highlight the diverse applications of EVs in treating various disorders. The modulation of EV content, including proteins, small RNAs, and lipids, emerges as a key strategy for tailoring therapeutic effects, showing promise for future developments in regenerative medicine and disease treatment.

Besides their use as therapeutics, EVs hold a great potential as therapeutic nano-biocarriers for drug delivery. Namely, EVs can encapsulate drugs or be decorated with specific ligands for targeted delivery. The pharmacokinetics of intrinsic and extrinsic EVs make them good candidates for drug delivery platforms owing to their in vivo tolerance and ability to cross biological barriers. Thus, different EV labeling and engineering strategies were developed to understand the in vivo pharmacokinetics of exogenously administered EVs. Labeling EVs can be performed with fluorescent dyes, radiotracers, or employing genetically engineered producer cells that express a reporter protein fused to the EV sorting. However, evident flaws of labeling using covalent conjugations include the alteration of the surface proteome of EVs, which affects their interactions with other proteins. Furthermore, these dyes can label non-vesicular proteins, which makes them imprecise. Additionally, EV engineering strategies, including the use of surface proteins like CD47, albumin, and polyethylene glycols (PEGs) to enhance circulation half-life, prevent clearance, and improve extrahepatic delivery, could also enhance the potential of EVs for drug delivery and targeting. Genetic engineering approaches also have drawbacks, such as the inability to label all EVs, as well as problems related with genetic engineering of producing cells. Additionally, the overexpression of specific EV-sorting proteins has the potential to alter EV biogenesis and/or proteome, impacting the biodistribution of EVs.

Pharmaceutical application of EVs in drug delivery is currently in the pre-clinical stage. EVs present challenges in designing suitable dosage forms for specific applications. Despite challenges, EVs demonstrate great potential in integrating small molecules, proteins, nucleic acids, and theranostics for therapeutic and diagnostic purposes. Being biologically derived, EVs offer complexity requirements ideal for nanomedicine. Importantly, patient-derived EVs hold promise for personalized medicine due to their biocompatibility and safety. From a pharmacokinetic point of view, EVs exhibit superior circulation time compared to LPs, enabling them to cross biological barriers effectively. Overcoming obstacles in clinical translation involves disclosing physicochemical properties, controlling drug release, employing advanced production techniques, ensuring storage stability, and fitting models for successful clinical trials. The exploration of LP-based dosage forms for various administration routes may pave the way for improved EV-based dosage forms in the future.

## Data Availability

Not applicable.
